# Genome-Wide Delineation of Natural Variation for Pod Shatter Resistance in *Brassica napus*

**DOI:** 10.1371/journal.pone.0101673

**Published:** 2014-07-09

**Authors:** Harsh Raman, Rosy Raman, Andrzej Kilian, Frank Detering, Jason Carling, Neil Coombes, Simon Diffey, Gururaj Kadkol, David Edwards, Margaret McCully, Pradeep Ruperao, Isobel A. P. Parkin, Jacqueline Batley, David J. Luckett, Neil Wratten

**Affiliations:** 1 Graham Centre for Agricultural Innovation (an alliance between NSW Department of Primary Industries and Charles Sturt University), Wagga Wagga Agricultural Institute, Wagga Wagga, NSW, Australia; 2 Diversity Arrays Technology Pty Ltd, University of Canberra, Bruce, ACT, Australia; 3 University of Wollongong, Wollongong, NSW, Australia; 4 NSW Department of Primary Industries, Tamworth Agricultural Institute, Tamworth, NSW, Australia; 5 Australian Centre for Plant Functional Genomic, School of Agriculture and Food Sciences, University of Queensland, St Lucia, QLD, Australia; 6 School of Plant Biology, University of Western Australia, Perth, WA, Australia; 7 CSIRO Division of Plant Industries, Canberra, ACT, Australia; 8 International Crops Research Institute for the Semi-Arid Tropics (ICRISAT), Hyderabad, Andhra Pradesh, India; 9 Agriculture and Agri-Food Canada, Saskatoon, Canada; 10 School of Agriculture and Food Sciences, University of Queensland, St Lucia, QLD, Australia; National Institute of Plant Genome Research, India

## Abstract

Resistance to pod shattering (shatter resistance) is a target trait for global rapeseed (canola, *Brassica napus* L.), improvement programs to minimise grain loss in the mature standing crop, and during windrowing and mechanical harvest. We describe the genetic basis of natural variation for shatter resistance in *B. napus* and show that several quantitative trait loci (QTL) control this trait. To identify loci underlying shatter resistance, we used a novel genotyping-by-sequencing approach DArT-Seq. QTL analysis detected a total of 12 significant QTL on chromosomes A03, A07, A09, C03, C04, C06, and C08; which jointly account for approximately 57% of the genotypic variation in shatter resistance. Through Genome-Wide Association Studies, we show that a large number of loci, including those that are involved in shattering in Arabidopsis, account for variation in shatter resistance in diverse *B. napus* germplasm. Our results indicate that genetic diversity for shatter resistance genes in *B. napus* is limited; many of the genes that might control this trait were not included during the natural creation of this species, or were not retained during the domestication and selection process. We speculate that valuable diversity for this trait was lost during the natural creation of *B. napus*. To improve shatter resistance, breeders will need to target the introduction of useful alleles especially from genotypes of other related species of *Brassica*, such as those that we have identified.

## Introduction

Resistance to the shattering of reproductive structures (shatter resistance), which reduces seed loss, is a key trait- that has been selected during crop domestication. Rapeseed (also known as canola), the world's third largest oilseed crop, (*Brassica napus* L. spp. *oleifera*, 2n = 4× = 38, genome A^n^A^n^C^n^C^n^) is an amphidiploid species of the eudicot family Brassicaceae, which originated at least 10,000 years ago as a result of spontaneous hybridization between turnip rape (*Brassica rapa* L.; genome A^r^A^r^, 2n = 2× = 20) and cabbage (*Brassica oleracea* L.; genome C°C^o^, 2n = 2× = 18), followed by chromosome doubling [1]. However, rapeseed was domesticated as an oilseed crop only 400–500 years ago [2]. Unlike the key cereal species, the total prevention of pod shattering and seed loss was not targeted for selection during the domestication of rapeseed. As a consequence, high levels of pod shattering still remain. This is a major bottleneck for commercial rapeseed production worldwide, as in that it can account for up to 50% yield loss [3].

The lineages of the two ancestral species *B. rapa and B. oleracea* diverged ∼3.7 million years ago (Mya) [4] from a single source [5], which itself diverged from the ‘model’ plant *Arabidopsis thaliana* L. approximately 20 Mya [6]–[9]. Yet despite this evolutionary divergence, the development and pod structure of *B. napus* is very similar to that of *Arabidopsis*, such that two pod valves, which enclose seeds, are joined together through a replum with valve margin cells (Figure S1). At maturity, these cells separate, thereby allowing the valve to detach from the replum releasing the seeds. Analysis of mutations in *Arabidopsis* has shown that genes encoding transcription factors, *SHATTERPROOF1* (*SHP1*), *SHATTERPROOF2* (*SHP2*), *NAC* (*NST1* and *NST3*), and the basic helix-loop-helix protein genes *INDEHISCENT* (*IND*), and *ALCATRAZ* (*ALC*) are involved in valve-margin development [10]–[18]. The *BEL1*-like homeobox gene *REPLUMLESS* (*RPL*) and the *FRUITFULL* (*FUL*) gene, which are expressed in valves, repress the expression of valve-margin identity genes [19]. Ecotypic expression analyses of *IND*, *PG* (Polygalacturonase) and *FUL* genes have shown their roles in regulating resistance to pod shatter in *B. oleracea, B. napus* and *B. juncea* [12], [15], [20].

Studies on other crops, such as rice, sorghum and wheat indicate a role for *SHAT1, Shattering1* (*Sh1*), *SH4*, and *RPL* genes in conferring natural variation in shatter resistance [21], [22]. For instance, a Single Nucleotide Polymorphism (SNP) in the promoter region of the *RPL* gene has been shown to be responsible for loss of shattering in rice [23]. A recent study showed that seed shattering in sorghum is controlled by a single gene *Sh1*, which encodes a *YABBY* transcription factor. Comparative analysis showed that similar gene orthologs (*OsSh1* in rice, and *ZmSh1* in maize) control shatter resistance in cereals [24]. The results of this study suggested that *Sh1* genes were under parallel selection during domestication. It is currently unknown whether domestication resulted in selection for similar genes across multiple species, including *B. napus* which conferred shatter resistance.

Natural genetic variation for shatter resistance has been identified in distant tribes of the Brassicaceae [25] and within significant oilseed crop species, such as *B. rapa*, *B. napus*, *B. juncea*, and *B. carinata* [26]–[28]. For example, a significant genetic variation for shatter resistance was reported in 13 *Brassica* accessions comprising three of *B. rapa* (B-46, DS-17-D, and Torch), four of *B. napus* (Isuzu, Midas, RU-1, and Wesroona), and six of *B. juncea* [28]. The two *B. rapa* accessions of Indian origin, DS-17-D and B-46 (which has four pod valves and incompletely developed replum), are highly shatter resistant, whereas the Canadian cultivar Torch is highly susceptible to pod shatter [29]. Genetic analysis of an F_2_ population derived from a cross between the parental lines DS-17-D and Torch showed that two recessive major genes, referred to as *sh1* and *sh2*, which have a dominant epistasis effect, confer shatter resistance. Two randomly amplified polymorphism DNA based markers, RAC-3_900_ and RX-7_1000_, were linked to recessive *sh1* and *sh2* alleles, and another SAC-20_1300_, was linked to both dominant *Sh1* and *Sh2* alleles [30]. Neither of these *Sh1* and *Sh2* loci has yet been mapped on the genetic linkage and or physical maps of *B. rapa*.

Earlier studies showed that a little variation in shatter resistance is present in current commercial breeding lines of *B. napus* [26], [28]. These studies evaluated a very limited number (7 to 12) of genotypes. Subsequent genetic analysis has revealed that additive gene effects contribute significantly to the phenotypic variation in shatter resistance [31]. Recently, one major quantitative trait locus that contributes 47% of the phenotypic variation, *psr1*, on chromosome A09 was mapped in an F_2_ population derived from Chinese parental lines of *B. napus* [32]. Wen *et al*. [33] identified 13 QTL for shatter resistance in a doubled haploid (DH) population derived from the cross H155/Qva. These QTL accounted for 38.6% to 49% of the phenotypic variation, depending on the growing environments. However, despite of these genetic analyses studies, the genetic bases of shatter resistance in the diverse *B. napus* germplasm have not been reported. There are several reasons for this lack of progress in the poor understanding of the shatter resistance trait. For example, this trait is strongly influenced by phenological attributes such as plant architecture, growing environment, physical conditions of pods during sampling and testing, and errors involved in estimating the shatter resistance [28], [34]. We report the extent of natural variation, and the genetic basis of shatter resistance in diverse germplasm of *B. napus*. We used a next generation sequencing based approach DArT-Seq and performed QTL and Genome-Wide Association Studies (GWAS) to decipher the genetic basis of shatter resistance in *B. napus*.

## Materials and Methods

### Mapping population

For QTL mapping, using microspore culture at Wagga Wagga, NSW, Australia we constructed a DH population comprising 126 lines from BLN2762/Surpass400. BLN2762 is an elite breeding line with reduced pod shattering that was developed in the NSW DPI canola germplasm development program. Surpass 400 is a commercial cultivar that has genes for resistance to blackleg disease, which is caused by the fungal pathogen *Leptosphaeria maculans* [35], but increased susceptibility to pod shattering (Andrew Easton, Pacific Seeds, Australia, personal communication). A panel of 186 diverse *Brassica* genotypes comprising 180 accessions of *B. napus*, two of *B. carinata* (ATC93184-1, ATC94044-1), three of *B. juncea* (CBJ001, Seetha, OasisCL), and one of *B. rapa* (Colt) (Table S1) was used for the molecular marker analysis. These accessions were obtained from the National Brassica Germplasm Improvement Program (NSW DPI, Wagga Wagga, Australia), the Australian Temperate Field Crops Collection, Horsham, Australia, and the USDA.

### DNA isolation

Young leaf tissue from different genotypes was collected for DNA extraction. DNA was extracted using a method described previously [36]. DNA was quantified using a Qubit dsDNA Broad Range kit (Invitrogen) and visualised for DNA quality on 1% TAE buffered agarose gels containing SYBR Green.

### Genotyping using DArT-Seq analysis

Similar to Diversity Arrays Technology (DArT) methods based on array hybridizations, the DArT-Seq technology was optimized for *Brassica* by selecting the most appropriate method for reducing the A^n^A^n^C^n^C^n^ genomic complexity (both the size of the representation and the fraction of a genome selected for assays). Four methods of reducing complexity were tested (data not presented) and the *Pst*I-*Mse*I method was selected. DNA samples were processed in digestion/ligation reactions principally as described previously [37], but replacing a single *Pst*I-compatible adaptor with two different adaptors corresponding to two different Restriction Enzyme (RE) overhangs. The *Pst*I-compatible adapter was designed to include the Illumina flowcell attachment sequence, sequencing primer sequence and staggered, varying length barcode region, similar to the sequence reported previously [30], [38]. The reverse adapter contained the flowcell attachment sequence and the *Mse*I-compatible overhang sequence. Only *Pst*I-*Mse*I fragments were effectively amplified in 30 rounds of PCR using the following reaction conditions: 94°C for 1 min, followed by 29 cycles of 94°C for 20 sec, ramp 2.4°/sec to 58°C, 58°C for 30 sec, ramp 2.4°C/sec to 72°C, 72°C for 45 sec. Finally, amplicons were held at 72°C for 7 min and then at 10°C. After PCR, equimolar amounts of amplification products from each sample of the 96-well microtiter plate were multiplexed and applied to c-Bot (Illumina) bridge PCR followed by sequencing on Illumina Hiseq2000. All amplicons were sequenced in a single lane. The sequencing (single read) was run for 77 cycles. Sequences generated from each lane were processed using proprietary DArT analytical pipelines. In the primary pipeline, the FASTQ files were first processed to filter away poor quality sequences; more stringent selection criteria (≥Phred pass score of 30) were applied to the barcode region than to the rest of the sequence. As a result, the assignments of the sequences to specific samples carried in the barcode split step were very reliable. Approximately 2,000,000 sequences per barcode/sample were identified and used in marker calling. Finally, identical sequences were collapsed into fastqcall files. These files were used in the secondary pipeline for DArT P/L's proprietary SNP and Presence/Absence Markers (PAM) calling algorithms (DArTsoft-seq). The analytical pipeline processed the sequence data. All polymorphic sequences of the DArT-Seq markers generated from the parental lines of the DH population from BLN2762/Surpass400, and from the diverse lines used in this study, were submitted to the Short Read Archive database under the bioproject (accession: PRJNA237043) of NCBI (http://www.ncbi.nlm.nih.gov/).

### Construction of a genetic linkage map

Molecular marker data that was based on SSR primer-pairs and traditional DArTs that was scored previously [36] was integrated with DArT-Seq markers (this study). Candidate gene specific primers for *SHP* (AF226865), *PG* (AC189368), *IND*, and *NST* (AC189597) were also analysed. Primer pairs for PCR analyses were identified using either the SSR Primer II or Primer 3 software programs. The sequences were: AC189597-forward 5′-ACAACAACAACAACAAC-3′ and reverse

5′-GAGAAGAAGAGGCATTCATT-3′ [targeting (ATG)_5_/(ATC)_6_ motifs of NAC SECONDARY WALL THICKENING PROMOTING FACTOR 1-*NST1* gene], and AC189368-1 forward 5′-GTTGGTAGCTCCCCAACAAA-3′ and reverse

5′-TGGTGATGAAGGTGATGATTG-3′. *SHP* specific-primers were: SHP1-00398-3 forward 5′-TCTTTGCTTTCTTGGTTTACT-3′ and reverse

5′-TCTTCCTTCTTCATTACTTGCT-3′, and SHP1-00925 forward 5′- GCTTGTTCCGATGCCGTT—3′ and reverse 5′-GAATGTCCCGAATCTGCC-3′. *IND* gene specific-primers were obtained from published sequences [15]. A linkage map was constructed using the package R/qtl [39], to compare results with those generated by the DArT P/L's mapping software OCDmap. Briefly, markers were binned, using a threshold of 0.14, and initial marker groups were ordered using the Lin-Kernighan heuristic TSP solver algorithm [40]. Data were cleaned up and errors were masked with threshold values of “H” = 8; “Missing Data” = 12; “Recombination” = 12. Error-masked data was then binned again with a threshold of 0.2 and re-ordered to produce the final map order. Recombination frequencies were converted to centiMorgan (cM) map distances using the Kosambi function.

### Genetic linkage analyses

The phenotypic data model developed for each trait (which was based on linear mixed-model technology) was used in the whole genome average interval mapping (WGAIM) approach to identify QTL associated with resistance to pod shattering [41] using the linkage map of the BLN2762/Surpass400 population. Empirical Best Linear Unbiased Predictions (eblups) from the phenotypic data model were used in a Statistical Machine Learning (SML) analysis [42] to compare the robustness of QTL detection between different algorithms. GWAS was performed using a SML approach with and without population structure using principal coordinates [43]. Principal coordinate analysis (PCO) was performed using all polymorphic markers. Genome-wide analysis was also performed to identify associations using a general linear model and mixed-model approach that accounts for population structure as a fixed effect and genetic relatedness as a random effect, as implemented in the Golden Helix SNP and Variation Suite version 7.7.8 (Golden Helix, Inc., Bozeman, MT, www.goldenhelix.com). Marker data were filtered and SNPs with minor allele frequency <0.05 were discarded. A total of 180 *B. napus* genotypes were used to identify common sequence variants involved in shatter resistance.

### Phenotypic analysis for pod strength

At maturity, 10 pods from five plants per genotype were collected from the middle portion of the main raceme and stored securely in capped plastic vials that contained a desiccant (silica) sachet, to prevent damage and stabilise moisture content. The strength (rupture energy = RE) of up to five individual pods from five random plants sampled from each genotype was measured using a pendulum apparatus that struck the pod with a known force and recorded the energy absorbed by the pod in shattering [44]. The pod length (PodLen) and rupture energy (RE) were measured for each pod. RELSQ was calculated as a measure of RE adjusted for variation in pod length (RE/(Podlen^2^)*1000) as described previously [28].

### Experimental design for phenotyping for pod strength

A total of 126 DH lines and their parental lines, BLN2762 and Surpass400, were grown in three field experiments. SHT11 was a partially replicated pot experiment with 72 duplicated lines and 56 unreplicated lines. The trial was arranged in a 4 row ×50 column array with duplicates split between 2×50 arrays. SHT12 was a two-replicate pot experiment arranged in a 4 row ×65 column array with replicates of 2×65 array. SHT12WW was a two-replicate field experiment arranged in a 15 row ×18 column array with replicates of 15×9 array. All experimental designs were generated using DiGGer [45].

A diversity set of 210 accessions, representing contemporary cultivars and elite lines from Australian and International programs: 197 of *B. napus*, six of *B. rapa* (AC-Sunshine, Yellow Sarson accession B-46, Brown Sarson accession DST-17-D, Colt, IB-5 and Torch), five of *B. juncea* (CBJ001, OasisCL, SaharaCL, Seetha and Urvashi) and two of *B. carinata* (ATC93184-1, ATC94044-1) was assembled. However, due to the unavailability of seeds of some lines, a subset of these accessions was used in different experiments, as shown in Table S1. The accessions were grown in outdoor pot and field experiments in 2010 and 2011 at the Wagga Wagga Agricultural Institute (New South Wales, Australia). The pot experiment conducted under Birdcage conditions (BIRDCAGE experiment) was originally arranged in two replicates on two benches in a glasshouse in a 4 row ×94 column array. The 2 row ×94 column array of pots on each bench was composed of 24 trays, each containing 1 row ×8 columns of pots. The pots were transferred to an outdoor birdcage enclosure until the end of the experiment using the same design. Among 178 genotypes raised to maturity, 159 genotypes were duplicated and 19 were unreplicated. Accessions in the SHT195 field experiment were arranged in two replicates in a 15 row ×26 column array, each replicate consisting of a 15×13 array. Data were available for two replicates of 184 genotypes and 8 unreplicated genotypes. The GD200 experiment was a two-replicate experiment in a 4 row ×100 column array, each replicate consisting of 2×100 array. RE was square-root transformed to stabilize variance in the linear mixed model analysis. The genotype effect was treated as a random factor. Broad sense heritability was calculated as described previously [46].

### Anatomical studies

Pods were collected at 35–40 days after anthesis. Hand sections were cut from fresh pod samples from the middle section of the pods (siliqua). Some of these were stained with Toluidine blue (pH 4.4). Others were observed for autofluorescence using a fluorescence microscope. Photographs were taken using a Zeiss Axiphot microscope for bright field fitted with a Sony Cyber-shot digital camera.

### Cluster analysis

DArT-Seq (SNP and *in silico* DArT) and non-DArT-Seq markers [36] (SSRs, and traditional DArT markers) and candidate-gene based markers for *SHP*, *PG*, *IND*, and *NST* (described under materials and methods) analysed across diverse genotypes of *Brassica* were used for cluster analysis using Gower's distance coefficient. Phylogenies were constructed using the hierarchical method, UPGMA.

### Physical (in silico) mapping of DArT-Seq marker sequences with the reference sequenced *B. rapa* and *B. oleracea* genomes

The newly discovered DArT-Seq marker sequences were aligned against both the sequenced scaffolds of *B. rapa* and contigs from *B. oleracea* (I. Parkin and A. Sharpe, unpublished data) using the Bowtie and local Blast implementation at DArT P/L. A significance threshold of E^-15^ was applied and the top three matches (chromosome and position), as well as the total number of significant alignments were recorded. DArT-Seq sequences that showed significant identities with the *B. rapa* genomic sequences were BLASTed against the C genome contigs in order to identify the orthologues. To map candidate genes involved in organ identity and pod shattering on *B. napus* genome, query sequences of *Arabidopsis* and *Brassica* species were aligned with the A and C genomic sequences. For comparative analysis of QTL regions, genetic and physical map positions were aligned and displayed graphically using the MapChart program.

## Results

### Generation of linkage map of *B. napus* through DArT-Seq Technology

We used a complexity reduction method to enrich genomic representations with single copy sequences and then performed next generation sequencing (NGS) of these representations using Illumina HiSeq2000 [37]. Thus, DArT-Seq is a new method of sequencing complexity reduced representations [47] that can also be used on the next-generation sequencing platforms [38], [48]. This pipeline just described uses a “reference sequence” constructed from sequences generated from *B. rapa*, *B. napus*, *B. juncea*, and *B. carinata* samples. Using as a basis the alignment of all tags for each target (library) against the reference, the pipeline identifies SNPs and PAMs using a number of technical parameters, which include the sequencing depth for each marker and their scoring reproducibility among technical replicates of libraries.

To test the efficacy of DArT-Seq technology and to identify QTL that confer resistance to pod shattering, we genotyped a DH population from a cross between BLN2762 and Surpass400, which resulted in the identification of 16,774 polymorphisms (3,041 SNPs and 13,733 PAMs). We integrated this dataset with 530 array-based DArT markers [36], [49], 112 simple sequence repeat (SSRs), and four candidate gene markers that were scored previously [36], and generated a linkage map with a total of 17,420 polymorphisms. These markers were distributed on all 19 linkage groups (Table S2; Figure S2). Several markers showed segregation distortion (Table S3). Among the DArT-Seq markers, the percentage of PAMs (78.8%) was greater than that of SNPs (17.5%). Of the SNPs, 1716 (71%) were transitions and 700 (29%) were transversions (Table S4), yielding a ratio of 2.45∶1. To confirm the genetic locations of DArT-Seq markers on the A^n^A^n^C^n^C^n^ genome, Illumina reads were aligned against both the sequenced scaffolds of A^r^A^r^ and contigs from C°C^o^ genome using the Bowtie and local Blast implementation at DArT P/L. A majority of these markers (69.1%) were aligned with the physical maps of A^r^A^r^ and C°C^o^ genomes. Overall the technology provided excellent genome coverage, due to the scanning of over 100,000 mostly low-copy sequences for DNA variation: and over 16,000 polymorphic markers were identified. Success was possible even in this population, which has relatively narrow genetic diversity between the parental lines (as shown here by our data).

### QTL analysis of natural variation in shatter resistance

**Figure 1:**
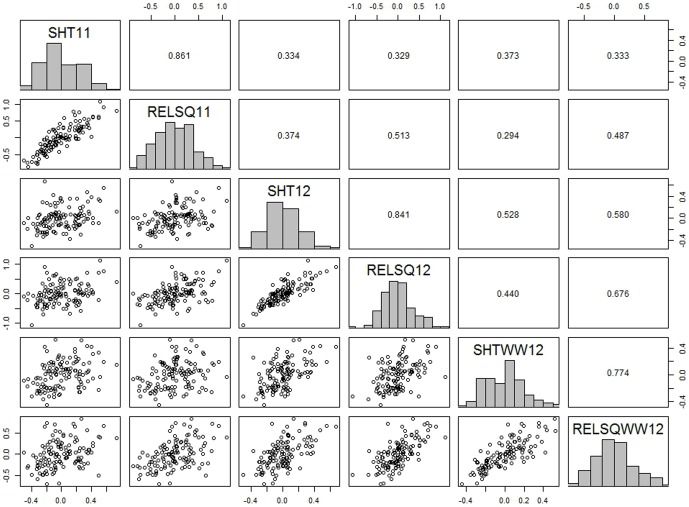
Distribution of shatter resistance, as measured with the pendulum test, among DH lines from the BLN2762/Surpass 400 population grown under three environments: experiment 1 (2011, screenhouse, SHT11); experiment 2 (2012, screenhouse, SHT12); and experiment 3 (2012, screenhouse, SHTWW12). Pair-plots of EBLUPS from DH lines and parental lines showing correlations are presented. Rupture energy (RE) was measured in mJ. Data from experiments SHT11, SHT12 and SHTWW12 were RE, whereas for experiments RELSQ11, RELSQ12 and RELSQWW12 the data were RE (adjusted for pod length) from the same lines.

To identify natural genetic variation for shatter resistance in *B. napus*, we used the same DH population, because BLN2762 differs from Surpass400 with respect to shatter resistance. We phenotyped the DH population for pod strength in 2011 and 2012, in three different experiments under field conditions. Two parameters for pod strength were measured– RE, and RELSQ. The approximate broad sense heritability (H^2^) values are given in Table 1. The H^2^ estimate for pod strength was very high, varying from 73.1% to 89.8% across environments (experiments). The ASReml [50] analysis of the phenotypes indicated a continuous and transgressive segregation, which suggested that several loci contribute to variation for pod strength in the BLN2762/Surpass400 DH population (Fig. 1). The two parameters of pod strength were found to be correlated positively (r = 0.77 to 0.86) within the same environment. However, correlations between environments (experiments) were moderate (r = 0.29 to 0.67). The predicted means of both parental lines and their DH progeny are given in Table S5.

**Table 1:** Significant QTL associated with resistance to pod shatter (pod strength or rupture energy) identified in a doubled haploid population derived from BLN2762/Surpass400

Trial	QTL	H^2^ (%)/C.V.	Chromosome	Marker Interval	LOD	Additive Effect	R^2^ (%)	Size (mJ)
*Rupture Energy*								
Expt. 1-2011	***Qrps.wwai-A03b***	84.3/15.6	**A03**	**3167032/3082606**	**2.39**	**BLN2762**	**10.4**	**0.10**
	***Qrps.wwai-A09b***		**A09**	**3089525** ***/*** **3155356**	**4.41**	**Surpass400**	**17.6**	**0.12**
	*Qrps.wwai-C04*		C04	3129479*/*3111779	3.46	Surpass400	12.9	0.11
Expt. 2-2012	***Qrps.wwai-C03***	**73.1/18.4**	**C03**	**3149536/3154228**	**3.47**	**BLN2762**	**17.0**	**0.10**
	***Qrps.wwai-C08b***		**C08**	**3124373/3142299**	**3.79**	**Surpass400**	**19.1**	**0.11**
Expt. 3-2012	***Qrps.wwai-A09b***	79.7/13.4	**A09**	**3155356/3104590**	**3.30**	**Surpass400**	**16.5**	**0.10**
	*Qrps.wwai-C08c*		C08	3117318/3162074	2.39	Surpass400	11.7	0.08
*#RELSQ*								
Expt. 1-2011	*Qrps.wwai-A03a*	89.8	A03	3145484/3095279	2.49	BLN2762	7.1	0.12
	***Qrps.wwai-A03b***		**A03**	**3167032/3082606**	**2.95**	**BLN2762**	**8.6**	**0.14**
	*Qrps.wwai-A09a*		A09	3118357/3126254	2.90	Surpass4000	8.9	0.14
	*Qrps.wwai-C06b*		C06	3170023/3110645	2.95	BLN2762	12.8	0.15
	*Qrps.wwai-C06a*		C06	3108912/3153168	3.99	BLN2762	12.8	0.17
	*Qrps.wwai-C08a*		C08	3134348/3174018	2.02	Surpass400	5.8	0.11
Expt. 2-2012								
	*Qrps.wwai-A07*	73.1	**A07**	3079412/3110084	**3.19**	Surpass400	**10.4**	**0.14**
	***Qrps.wwai-C03***		**C03**	**3149536/3154228**	**5.14**	**BLN2762**	**17.7**	**0.19**
	***Qrps.wwai-C08b***		**C08**	**3142299/3112431**	**8.25**	**Surpass400**	**28.9**	**0.24**
Expt. 3-2012								
	***Qrps.wwai-C03***	**84.2**	**C03**	**3154228/3114475**	**2.80**	**BLN2762**	**11.6**	**0.13**
	***Qrps.wwai-C08b***		**C08**	**3112431/3103276**	**4.80**	**Surpass400**	**20.1**	**0.17**

Flanking markers that show the LOD score ≥2 are only shown, the additive effect refers to the parental allele that showed an increased effect and, the percentage of genotypic variation (R^2^) explained, and size of QTL effect (pod strength in millijules -mJ). The QTL analysis was carried out using a whole genome average interval mapping approach in R software. Bold letters indicate consistent QTL detected across different experiments/Pod shatter resistance attributes. H^2^: Broad sense heritability, C.V: Coefficient of variation. #: Estimations of RELSQ are natural logarithmic transformation therefore C. V. values are not provided.

Whole genome average interval mapping (WGAIM), which has been shown to be superior to composite interval mapping with respect to detecting genuine QTL [41], identified 12 significant QTL (P≤0.002) on chromosomes A03, A07, A09, C03, C04, C06, and C08. Taken together, these QTL jointly explained a total of 57% of the genotypic variation for pod strength (Table 1). Amongst these QTL, *Qrps.wwai-A03b, Qrps.wwai-A09, Qrps.wwai-C03*, and *Qrps.wwai-C08* on homoeologous chromosomes A03/C03 and A09/C08, were consistent across at least two of the three phenotyping experiments. The most significant QTL, *Qrps.wwai-C8b* with a LOD score of 8.25 (R^2^ = 28.9%), was located on chromosome C08 between DArT-Seq markers 3142299 and 3112431. BLN2762 contributed favourable alleles for shatter resistance at loci on chromosomes A03 and C03, whereas Surpass400 contributed favourable alleles on homoeologous chromosomes A09 and C08 (Table 1). To test the robustness of QTL detection, we used a Statistical Machine Learning (SML) method [42]. Some of the QTL and their effects were consistent between WGAIM and SML, although other significantly associated genomic regions (QTL: up to 39 markers with P≤0.001) were also identified (Table S6).

### Natural variation for shatter resistance in *Brassica*

**Figure 2:**
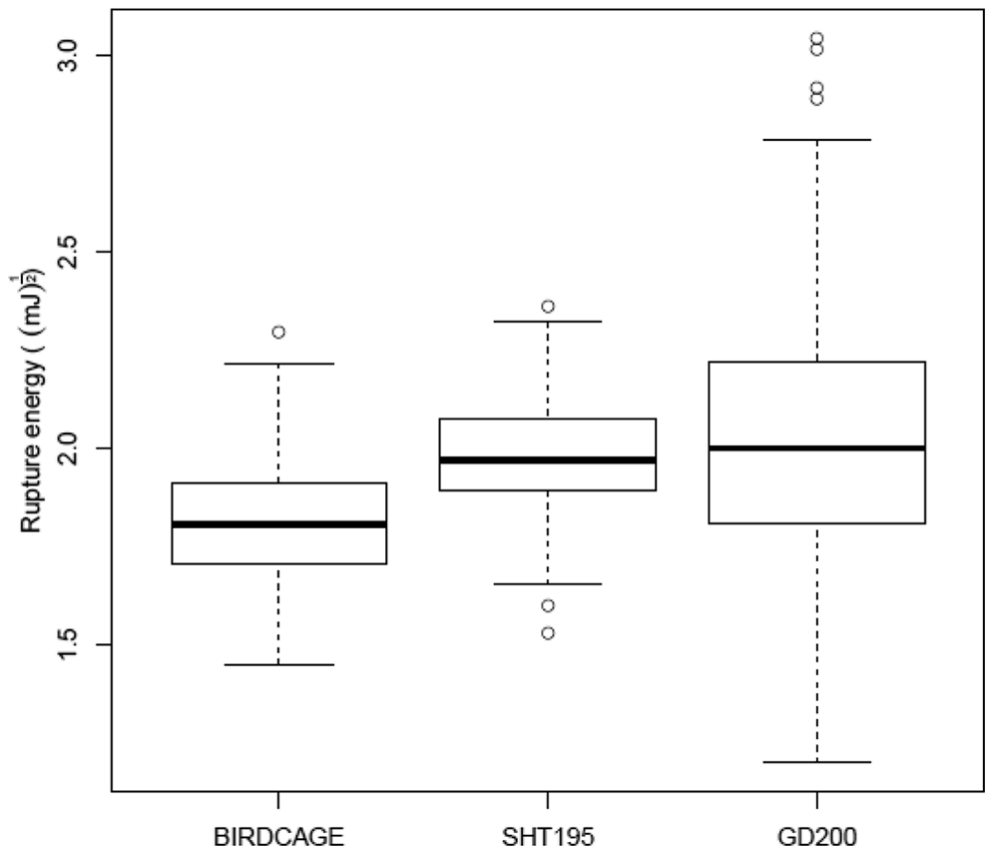
Box-plot showing variation for resistance to pod shatter in population of DHs from BLN2762/Surpass400, grown under three different environments: Experiment 1, 2010, Birdcage; Experiment 2, 2011, Birdcage; and Experiment 3, 2011, Field conditions).

To investigate the extent of allelic richness and to delineate genomic regions that contribute significantly to shatter resistance, we conducted three experiments under bird-cage and field conditions on 210 accessions of *B. napus*, *B. rapa*, *B. juncea*, and *B. carinata* (Table S1) to further evaluate the pod strength of the diversity panel. Variation in shatter resistance across different experiments was observed (Fig. 2). Pod strength varied from 2.09 mJ to 5.28 mJ and from 2.34 mJ to 5.58 mJ, in the bird-cage and field experiments, respectively. Pod strength measurements from both the birdcage and field experiments in 2011 and 2012 (Figure S3) were correlated (Pearson correlation coefficient = 0.5 to 0.6). There was a significant variation for pod strength due to genotype. The results of variance components analysis for RE (Table S7) showed positive covariate values that indicated that RE increases with pod length. The covariate was significant in all cases (P<.001). The percentage of variability associated with genotype ranged from 16.6% to 27% of the total variability.

### Association mapping of shatter resistance loci

A diversity panel was genotyped with: a DArT array [49], a set of 22 selective SSR markers that showed significant associations with other traits of agronomic importance (resistance to blackleg, and flowering time) [46], [51]–[53], candidate genes involved in shatter resistance (*SHP1*, *IND* and *NST*); and a DArT-Seq GBS pipeline. The resulting dataset comprised 89,618 polymorphic markers (37,245 SNPs and 52,373 PAMs) across the different accessions (Table S8). The call rate of SNPs ranged from 80% to 100% with an average of 96.7%. Scoring reproducibility was 99.5% for all selected markers. Polymorphic information content varied from 0.04 to 0.49, with an average of 0.2. To ensure proper classification of the germplasm utilised, we carried out a phylogenetic analysis, which revealed, as anticipated, that *B. carinata*, *B. juncea* and *B. rapa* are members of different clades than *B. napus* (Figure S4). The cluster V consisted of a large number of accessions of *B. napus*, which were grouped according to their pedigrees and their geographical origins (e.g. China or Australia). Many lines with the ‘Roy’ suffix were grouped in a distinct cluster; this might be because they were derived from interspecific crosses between *B. napus* and *B. juncea*. Principal coordinate analysis revealed the sub-population structure among the diverse lines representing different Brassica species. The first two principal coordinates, PCO1 and PCO2 explained 82% and 10%, respectively of the genetic variation among different *B. carinata*, *B. juncea*, *B. rapa*, and *B. napus* genotypes investigated (Figure S4; Figure S5a).

**Figure 3:**
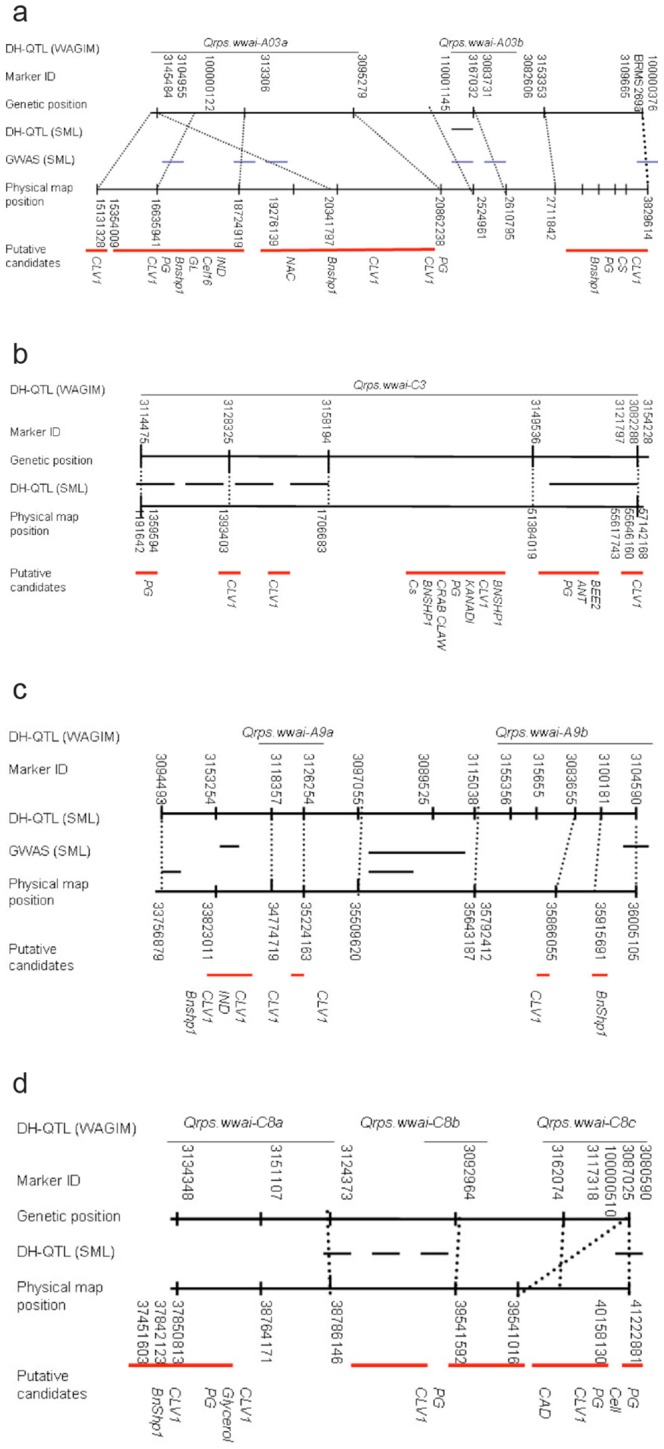
QTL detected with linkage (whole average genome interval mapping – WGAIM [DH-QTL], statistical machine learning-SML [DH-QTL], and genome-wide association analysis [GWAS] in *Brassica napus* germplasm. Marker sequences were aligned with the sequenced genomes of *B. rapa* and *B. oleracea* and their physical positions are shown *with dotted lines* (Tables S1& S4). Putative candidate genes (marked with red lines) that were localised within the physical map intervals are listed. Only QTL consistent across environments are shown (Table 1): a = *Qrps.wwai-A03a* and *Qrps.wwai-A03b*; b = *Qrps.wwai-C03*; c = *Qrps.wwai-A09a*, and *Qrps.wwai-A09; b* and d = *Qrps.wwai-C08a*, *Qrps.wwai-C08b* and *Qrps.wwai-C08c*.

GWAS was then conducted to determine different networks of alleles for pod strength by exploiting historical recombination among loci exclusively in 180 *B. napus* genotypes. The top two components, PCO1 and PCO2 explained 62.2% of the genetic variation in *B. napus* genotypes (Fig. S5b). The high percentage of variance captured by the first two principal coordinates indicates significant differentiation among the genotypes, with winter- versus spring-type separation being a major division between the genotypes. To reduce spurious association due to population structure, we used both coordinates PCO1 (42.5%) and PCO2 (19.7%) as cofactors [43] in association analysis. In addition, we used pod length as a covariate, because the diverse *B. napus* lines showed significant variation for this trait (Table **S7**). The GWAS with SML method revealed several significant associations between the pod strength and genotypic marker data; 97 to 111 markers showed significant statistical associations (p<0.001) with shatter resistance (strong signals), whereas medium-strength signals were detected for 131 marker loci at p values ranging from 0.01 to 0.002 (Table S9). Several markers associated with shatter resistance were common between the GWAS and the linkage analysis experiments (Table S9). The GWAS peaks explained by 45 markers on chromosomes A03 (1 marker), A07 (1 marker), A09 (5 markers), C03 (16 markers), C06 (2 markers) and C08 (20 markers) were consistent with the linkage analysis in the BLN2762/Surpass400 population (Fig. 3, Table S10). Several markers that were identified with the SML approach were similar to those that were identified with GoldenHelix software.

### Analysis of candidate genes in mapping intervals

The complete genome assembly of *B. napus* is not yet available in the public domain. To identify and characterise putative candidate genes for pod shatter resistance, and to gain an insight into their organisation, we utilised the sequenced A^r^A^r^ and C°C^o^ subgenomes of the ancestral species of *B. napus*. We aligned all the marker sequences that were polymorphic between BLN2762 and Surpass400 with physical scaffolds of the reference A and C genomes (comprising 283.8 Mb of A^r^A^r^ and 486.6 Mb of C°C^o^, genomes, respectively) and looked for co-localisation with genes known to be involved in pod shatter in *Arabidopsis* and *Brassica* species. A good colinearity between genetic and physical map positions was observed (Figure S6). However, several genomic regions showed genomic rearrangements, including a well-known reciprocal inversion between chromosomes A07 and C06 (Figure S7) [54]. This was verified by aligning sequenced A07 chromosome-specific DArT clones with C06 scaffold sequences.

On the basis of alignment of the genetic position of QTL for resistance to pod shatter with the physical map position of marker sequences, at least eight candidate genes: *FULL* (*AGL8*), *CLV1-like receptor kinase* (*CLAVATA*, *AAP49010.1, B. napus*), *AGAMOUS-Like 15-*(*AGL15, ABD77425.1 B. napus*), *SHP1*(*B. napus, AAK00646.1*), *RPL*, *HECTATE* (NM_121012.1, *A. thaliana*), *IND* (CAZ66758.1), *CELLULASE 16*, *AP2-like ethylene-responsive transcription factor* (*ANT*) and *PG,* were identified as being located within distinct marker ‘clusters’ or in the vicinity of organ identity genes of Arabidopsis (within 100 kb) such as, *AGAMOUS, CLAVATA, CRAB CLAW, DELLA*, and *KANANDI* (Table S10, Fig 3). Some DArT-Seq markers were mapped in close proximity to shatter resistance genes, for example locus 100000122 (*Qrps.wwai-A3a*), which was mapped 2.1 kb apart from the *PG* gene on chromosome A03. Likewise, markers 3169069 and 3109148 underlying the *Qrps.wwai-C08a* were mapped within 1.6 kb of the *PG* gene on chromosome C08 (Table S10).

To determine whether the same gene(s) underlie the QTL regions detected on homoeologous A03/C03 and A09/C08 chromosomes of the A_r_A_r_ and C_o_C_o_ genomes, we compared their genomic organisation (Table 1). Both QTL regions *Qrps.wwai-A03b* and *Qrps.wwai-C03*, localised on chromosomes A03/C03, showed colinearity, and possessed the *BnSHP1* (*SHATTERPROOF* gene in *B. napus*) gene (Figure. S6). Likewise, *Qrps.wwai-A09b* and *Qrps.wwai-C08b,* localised on chromosomes A09 and C08, respectively, showed significant homoeology with each other, although some regions showed rearrangements that disrupted their colinearity. Both these genomic regions also harbored the *BnSHP1* gene. The QTL *Qrps.wwai-A07* and *Qrps.wwai-C06* also included *BnSHP1*, although they were not detected consistently across environments.

The *SHP* gene was detected within the QTL intervals underlying shatter resistance in our LD and GWAS studies (Table 1), and has been described as a key gene regulating shatter resistance in *Arabidopsis* and other species [11], [19], we then further analysed its allelic variation in 126 DH lines using *SHP* gene-specific markers. Three *SHP1* loci, two of them designated as *Shp-1000398-3b* and *Shp-1000398-3c*, were located within 1cM of each other on chromosome A07, and one *Shp-100925* locus was mapped on chromosome A09 (Table S3). *In silico* mapping of *SHP* genes confirmed that corresponding copies are present in the A07 and A09 sequences of the *B. rapa* genome (Table S10). In addition, we mapped a marker specific to the *IND* gene (HB416515) in the same set of DH lines. The *IND* marker showed a distorted segregation ratio (2×BLN2762 alleles: 1×Surpass400 alleles), and was mapped on chromosome A03.

### Anatomical analysis of pod structures

**Figure 4:**
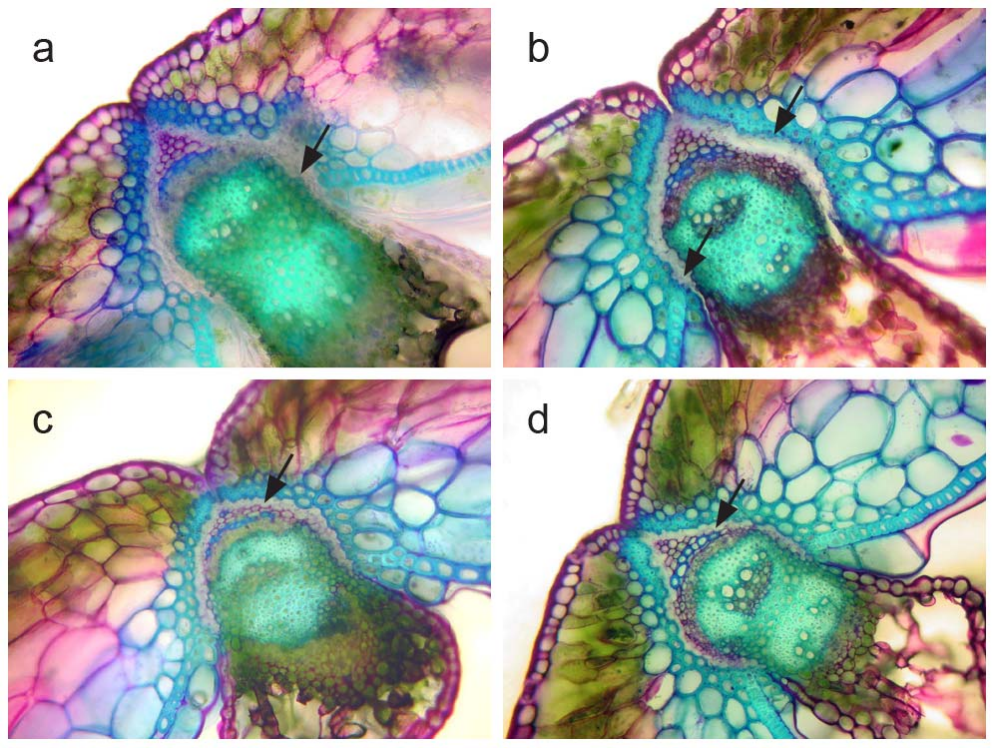
Anatomical differences among four exemplar haplotypes from the DH population derived from BLN2762/Surpass400. *a* = DH line 6668 is a haplotype with *Shp*(B) and *Ind*(S) alleles, b = DH line 6823 is a haplotype with *Shp*(B) and *Ind*(B) alleles, c = DH line 7128 has *Shp*(S) and *Ind*(B) alleles, and d = DH line 7124 has *Shp*(S) and *Ind*(S) alleles. Alleles B and S given in parentheses represent the parental donor lines of the DH population. Arrows show marked structural differences among haplotypes.

We analysed the anatomical pod structure of 32 DH lines from the BLN2762/Surpass400 population, using Toluidine Blue staining. These lines represented four haplotypes with different *SHP1* and *IND* alleles (Table S11). The DH lines that had BLN2762 alleles at *IND* and *SHP* were anatomically different from those that had the corresponding Surpass400 alleles (Fig. 4). Compared to other haplotypes, the structure of the replum-valve junctions revealed marked differences in lignification (cellulose/hemicellulose rich cell layers) and the presence of a conspicuous abscission layer between the valve cell and replum cell junction in haplotypes with the *SHP* gene from Surpass400. DH genotypes carrying favourable alleles at the QTL regions (Table 1) showed that genotypes with favourable alleles had greater pod strength than those without such alleles (Table S12). Lines carrying *SHP* marker alleles had greater pod strength.

## Discussion

### Multigenic inheritance of shatter resistance in *B. napus*

In the study reported herein, we investigated natural variation and mined alleles that are involved in pod shatter resistance in *B. napus*. A large number of loci involved in shatter resistance were detected, in contrast to the small number of genes that have been reported previously in *B. rapa, B. napus* and several cereal crops [24], [30], [55]. This is most likely due to the quantitative inheritance of shatter resistance in *B. napus* and the large, complex gene network involved in the diverse germplasm that we investigated. Moreover, the approaches used in the current study differ greatly from those used in earlier work and allowed a more comprehensive examination of shatter resistance. Like classical quantitative traits, pod shattering was influenced by the environment: only moderate broad sense heritability values were observed, and rupture energy scores measured over different experiments and seasons showed only moderate correlation (Fig. 1, Table 1). Transgressive segregation evident in the BLN2762/Surpass400 population suggested that positive useful alleles were contributed by both parents and that breeding for this trait can be successful.

### Localisation of shatter resistance loci on the *B. napus* genome

We located QTL for shatter resistance on three homoeologous chromosomes: A03/C03, A09/C08 and A07/C06. A major QTL, *Qrps.wwai-C8b* (R^2^ = ∼29%) was identified on chromosome C08. A recent study examined shatter resistance in a *B. napus* DH population derived from Chinese parental lines and detected 13 QTL (R^2^ = 3.4% to 10.2%, LOD = 2.10 to 4.7), on chromosomes A01, A04, A07, A08, C05, and C08 that accounted for up to 49% of the variation in resistance [33]. In a second study, genetic analysis of bulks consisting of shatter-resistant and shatter-susceptible lines of an F_2_ population derived from Chinese parental lines also showed quantitative inheritance of shatter resistance and one major QTL contributing 47% of variation, *psr1*, on chromosome A09 was identified [32]. These studies suggest that at least one QTL localised on homoeologous chromosome A09/C08 is present consistently across populations originated from Australia and China. However, involvement of those QTL in diverse *B. napus* germplasm has not been shown in previous studies. The majority of markers explaining the significant allelic effects for shatter resistance in the BLN2762/Surpass400 population were localised within ∼200 kB regions that contain candidate functional genes that are involved in pod shattering in *Brassica*, *Arabidopsis, Medicago* and cereal crops (Table S10). Our QTL and GWAS analyses showed clearly that several genes control shatter resistance in *B. napus* germplasm. One of them, *BnSHP1*, was detected consistently across different environments and underlies genetic variation for pod shattering at all QTL that were detected on the above three homoeologous chromosomes as well in diverse germplasm.

While we have not demonstrated the causative nature of allelic variation, their candidacy in conferring shatter resistance has already been tested via ecotypic expression in *Arabidopsis* and other *Brassica species*, including *B. napus*, and *B. juncea* [11], [12], [19], [20]. Given that the family of genes implicated in floral identity and shatter resistance occurs in multiple copies in both the diploid A and C genomes of *B. rapa* and *B. oleracea*, respectively (Table S10), demonstrating the functions of those alleles in conferring shatter resistance in the allotetraploid *B. napus* is a challenging exercise. For example, in rice and Arabidopsis, up to 70 copies of the *PG* gene, which are known to be involved in pod shattering have been predicted [16]. As the diploid species *B. rapa* and *B. oleracea* originated from the whole genome triplication of a common hexaploid ancestor, amphidiploid *B. napus* is expected to possess even more copies; and the organisation of shatter resistance genes will be complicated further by genome fractionation and sub-fragmentation over the past 10, 000 years. BLASTP analysis showed that several copies of genes involved in shatter resistance and organ identity exist in the *B. napus* genome and often are clustered in certain regions (Table S10). Intense selection pressure may have prompted gene family expansion in *B. napus*, as compared to its ancestors since its divergence from Arabidopsis. Another possible role of these genes may be in regulating other plant developmental processes. Some of the markers that showed significant association with shatter resistance in the BLN2762/Surpass400 population, and in a diversity panel, coincided with QTL (within 3 cM) associated with yield-related traits, such as seed number, pod number, seed weight, biomass production, seed yield, seed oil content, and flowering time, which were localised previously on chromosomes A01, A03, A09, C02, and C07 in the mapping populations derived from Tapidor/Ningyou7 and Skipton/Ag-Spectrum [46], [56], [57]. This could be attributed to pleiotropic effects or the presence of closely-linked genes involved in flower and pod development.

### Comparison between linkage (QTL) and association mapping (GWAS)

In the study reported herein, we used both a biparental population and a diverse germplasm panel in order to detect diverse favourable alleles for shatter resistance. A major drawback of the QTL interval mapping procedure has been the detection of large genetic (marker) intervals and a low density of genome-wide markers, which makes it difficult to determine the best candidate(s) for the causally operative genes [58]. In this study, we used over 17,000 genome-wide markers to map QTL. However, due to the smaller size of the DH population, we were unable to determine precisely the genetic locations of most of the co-segregating markers. Despite these difficulties, many co-segregating markers were mapped successfully in distinct positions on the physical map (Table S10). Smaller populations often lead to low resolution of genome mapping due to the limited detection of recombination events. High resolution mapping of individual QTL is required in order to (i) characterise QTL comprehensively, (ii) identify Quantitative Trait Nucleotides (QTNs) [59] causing phenotypic diversity for shatter resistance, and (iii) understand natural selection that occurs at these QTNs. Biparental populations allow the detection of two alleles and have strong statistical power, but they provide low genetic resolution if the population is small, as in this study. In contrast, GWAS can detect several functionally diverse alleles per locus in an unstructured population and provides high-resolution mapping. Therefore, our approach is useful for detecting genome-wide markers associated with shatter resistance, as it benefits from both classical-linkage and association-mapping strategies.

Several methods have been used for GWAS, for example PCA/PCO analyses using mixed linear models, multi-trait mixed-models and multi-locus mixed-models (MLMM) [43], [60], [61]. Previous studies concluded that no single GWAS method (based on a general/mixed linear model) is sufficient to unravel the genetic complexity underlying natural variation in crops [62], [63], because the efficacy of these methods is affected by population structure, kinship and allele frequency [64], [65]. Similar observations were made in the present study. In *B. napus*, we found a very narrow range of variation and detected markers with only low to moderate allelic effects for shatter resistance. The narrow range of genetic variation detected among commercially released *B. napus* varieties may be due to differences at certain QTNs that have contributed to natural variation for shatter resistance. Both traditional and more modern breeders, whilst wishing to maximise shatter resistance, may have unintentionally retained genes for shatter susceptibility due to their inability to select reliably multiple recessive alleles, and this may have contributed to the complex genetic network of shatter resistance genes found in current *B. napus* cultivars.

GWAS detects historical recombination in the germplasm, and identifies the common allele variants that contribute to phenotypic variation seen between genetically diverse lines. In the present study, the detection of strong signals accounting for major allelic effects via genome-wide marker-pod association with shattering may have been limited, due to the low allele frequency of loci involved in pod shattering in certain lineages (subpopulations). For example, in one lineage that we used, the Australian cultivar Surpass400 (very susceptible to pod shattering, derived from crossing *B. rapa* ssp. s*ylvestris* with *B. napus*), only four genetically-related cultivars (Surpass501TT, Surpass402CL, Surpass603CL and Hyola60) were present in the germplasm set.

### Analysis of natural variation for shatter resistance using DArT-Seq

DArT-Seq technology has addressed the major challenge of applying NGS technologies, as the complexity reduction method enabled us to sieve the complex genome of our polyploid plant species (*B. napus*) and identify 89,618 SNPs and PAMs (Table S8) in the absence of a reference sequenced genome. It is important to stress that the presence/absence markers do not correspond to Presence/Absence Variations (PAVs) (sequences really absent from the genome) but rather indicate the presence/absence of those sequences in genomic representations. In this regard, the presence-absence markers are analogous to DArT markers from microarray platforms and are often referred to as silico-DArTs, because they are extracted *in silico* from sequence data rather than from the presence/absence of a hybridization signal on DArT arrays. To distinguish between the presence/absence based on genetic and epi-genetic (methylation) factors, and absence due to under-sampling of a particular sequence in the representation of a given sample, the pipeline works by applying several filters similar to those employed in SNP calling (based on sequencing depth and technical reproducibility).

Our analyses showed that a DArT-Seq approach based on genome complexity reduction with endonucleases, coupled with multiplexing with barcodes, is suitable for deciphering loci that underlie a quantitative complex trait (pod shattering) in the amphidiploid genome of *B. napus* and for characterising the genome basis of the loci that are responsible. A DArT-Seq pipeline can be deployed to generate very dense linkage maps, suitable for molecular diversity analysis, QTL detection, and GWAS. Therefore, it can be used as an alternative to standard -fixed sequence approaches, such as the 60K SNP Infinium array. In addition, it does not suffer from ascertainment bias that is typical of such arrays, which is particularly important when analysing diverse germplasm, including wild germplasm. A high-density genetic linkage map based on sequenced markers, and their alignment with ancestral genome scaffolds, provides a reference for studying genome biology, comparative genomics analysis, and genomic exchange via introgression, as well as for predicting total breeding and genetic values for traits of agricultural significance, such as pod shattering [66]. Genome-wide marker-based selection will enable breeders to increase the selection efficiency for improved resistance to pod shattering and the other quantitative traits segregating in this diverse germplasm. It has been reported that the polyploid nature of *B. napus* interferes with both SNP discovery and high-throughput SNP assay technologies [67]. The sequence of a specific GBS marker locus can be used directly for genotyping individuals with designed PCR based markers. Unlike traditional DNA-hybridisation-based DArT [36], DArT-Seq is based on sequences of genomic representations. In addition, it enables the detection of heterozygotes at individual SNP marker loci, which is a valuable feature in improving selection efficiency in the early generations of breeding programs.

### Expansion of natural genetic variation for pod shatter resistance in *B. napus*

Although there was limited natural variation for shatter resistance in the *B. napus* germplasm that we investigated, it was useful for detecting and mapping associated loci. A lack of adequate genetic variability for breeding can be compensated for by introgressing genes from genetically diverse genotypes; this strategy may even produce superior genotypes by diversifying nuclear and cytoplasmic gene combinations. Our results on phenotypic, and phylogenetic analyses showed that alleles responsible for higher levels of shatter resistance exist in related *Brassica* species, such as in *B. carinata* (ATC93184-1, ATC94044-1), *B. rapa* (AC-Sunshine, B46 and DST17D), and *B. juncea* (CBJ001, SaharaCL, Seetha and Urvashi). However, such alleles may have been lost, during intensive selection, due to domestication bottlenecks, or due to linkage drag of undesirable alleles. The precise cause has not yet been determined. It is also possible that favourable allele combinations for shatter resistance were not present in the ancestral genotypes of *B. rapa* and *B. oleracea* that were actually involved in the hybridisation events that gave rise to *B. napus*; thus, canola may have evolved as a shatter susceptible crop in nature. Shatter resistance has been identified in 20 more distant relatives of the Brassicaceae including the species *Lepidium appelianum* [25]. The introgression of shatter resistance genes from different members of the Brassiceae (*B. rapa*, *B. carinata*, *B. juncea*, and *Raphanus sativa*) has been accomplished previously [34], [68], [69]. This suggests that genomes within Brassicaceae are plastic in evolution and amenable to further genetic manipulation via wide-hybridisation strategies. We have performed interspecific hybridisation to introgress alleles for shatter resistance from *B. rapa* accession B-46 into *B. napus* cv. Midas. Genetic analysis of an F_2_ population showed that the level of shatter resistance in Midas could be improved up to 12 times (Table S13). Hybrid-derivatives having A^r^A^r^C^n^C^n^ subgenomes are currently being tested for their agronomic performance. It is likely that many hybrid derivatives will have *B. rapa* genes that may not be desirable due to chromosomal rearrangements. Those allelic effects can be eliminated by accelerated backcrossing and using molecular markers. The suggested strategy for interspecific hybridisation will also allow the broadening of the genetic base of canola, leading to more efficient and fruitful breeding programs.

In conclusion, we observed a limited genetic variation for shatter resistance in *B. napus*. On the basis of our results, we speculate that valuable diversity for shatter resistance was lost during the natural creation of *B. napus*. If we are correct, breeders will need to embark on the targeted introduction of useful alleles from genotypes of other related species of *Brassica*. Our results showed that DArT-Seq is a suitable platform for genetic linkage map construction, QTL detection, GWAS analysis, molecular diversity analysis, and comparative analyses of shatter resistance in the polyploid genome of *Brassica*. In our study, at least three homoeologous genomic regions on chromosomes A03/C03, A09/C08 and A07/C06 that are associated with shatter resistance were identified via both linkage and genome-wide association approaches. Both these approaches to mapping enabled a comprehensive analysis of the genetic bases in natural variation for shatter resistance and confirmed the existence of consistent QTL across different environments and experiments (Table 1). Several markers, including some within the candidate functional genes involved in pod shattering in *Brassica*, *Arabidopsis*, *Medicago* and cereal crops (Table S10), such as *BnShp1*, were identified within ∼200 kB regions in a BLN2762/Surpass400 mapping population. The gene-specific molecular markers, including Shp-1000398-3b, Shp-1000398-3c and Shp-100925 (Table S3) provide a simple and effective tool for accelerating the selection efficiency of favourable alleles for shatter resistance in the practical breeding of *B. napus*. We also identified a suite of markers associated with shatter resistance in diverse germplasm accessions via GWAS, which provide a valuable dataset for genomics-assisted breeding in *B. napus*.

## Supporting Information

Figure S1 **Transverse section of the** ***B. carinata*** **accession ATC90239 pod at 40 days after anthesis visualised under fluorescence.**
(PDF)

Figure S2 **Mapping of DArT-Seq and non-DArT-Seq markers in relation to their recombination fractions and physical map positions on A and C genomes of** ***B. rapa*** **and** ***B. oleracea*** **, respectively.**
(PDF)

Figure S3 **Relationship between rupture energy among ∼200 diverse genotypes.** (A: BIRDCAGE and FIELD experiments, *p* value = 4.44e-16, r = 0.57; B: SHT and GD experiments, *p* value = 2.22e-16, r = 0.55, and C: BIRDCAGE and FIELD experiments, *p* value = 8.88E-15, r = 0.55). ‘r’ indicates Pearson's product-moment correlation.
(DOC)

Figure S4 **Phylogenetic analysis of DArT-Seq and non-DArT markers from different species of** ***Brassica*** **.** The *sidebars* indicate the clades of different cultivars/species. The tree was constructed by the UPGMA method with Gower's distance.
(DOC)

Figure S5 **Principal coordinates analysis revealing overall genetic variation present in the genetic data of the diversity panel.** The top 10 coordinates are shown in the bottom right panel along with the proportion of variance explained abbreviated as PAVE, on the y-axis. (a) PCO plots of first three axis (x, y and z) of *B. napus*, *B. rapa*, *B. carinata* and *B. juncea* genotypes and (b) PCO plots of first three axis (x, y and z) of *B. napus*.
(PDF)

Figure S6 **Comparative analysis of marker intervals underlying QTL for shatter resistance on homoeologous chromosomes (a) A08/C08, (b) A03/C03 and A07/C06 in a** ***B. napus*** **DH population from BLN2762/Surpass400.** Map positions are given to the *left* of the linkage groups (genetic distances are given in cM whereas, physical map distances are given in fractions (1/1,000,000^th^ of the actual coordinates) of the *B. rapa* and *B. oleracea* scaffolds. Locus names are listed to the *right*. The QTL regions are marked with vertical bars to the *left*. Homologues are joined with *solid* lines between linkage groups. Organ identity and shatter resistance genes are given in *italics*. Candidate genes underlying the QTL (Table 1) are in *bold*. Query sequences were aligned with the genome scaffolds of *B. rapa* (A_r_A_r_ genome) and *B. oleracea* (C_o_C_o_ genome) and subsequently graphically represented using MapChart. QTL regions are connected with dotted lines.
(DOC)

Figure S7 **Homoeology between chromosomes A07 and C06 based on DArT sequences.** Homologues are shown with solid lines.
(DOC)

Table S1 **List of genotypes, their country of origin, and species used for genetic diversity analysis.**
(DOC)

Table S2 **Salient features of the genetic linkage map of a DH population from BLN2762/Surpass400.**
(XLSX)

Table S3 **DArT-Seq and non-DArT-Seq markers that showed distorted segregation ratio within the BLN2762/Surpass400 population.** Calculated *p* is the *p*-value associated with the test for segregation distortion.
(XLSM)

Table S4 **Summary statistics of DArT-Seq and non-DArT-Seq markers (SSR, STS, traditional DArTs labelled with brPb-suffix) segregating in a DH population from BLN2762/Surpass400.**
(XLS)

Table S5 **Predicted means of the parental lines of BLN2762/Surpass400 DH population used for phenotyping.** Frequency distribution of DH lines is shown in Figure 1. Transformations are square-root of shatter and natural logarithm of RELSQ.
(DOC)

Table S6 **GWAS analysis showing molecular markers associated with shatter resistance in the diverse set of Brassica genotypes using Statistical Learning Machine method.** Highlighted markers are significantly associated with pod strength at *P* = 0.001. Physical map position '0' indicates no significant hit was found between query (GBS-Seq/DArT marker sequence) and the reference A^r^A^r^ and C°C^o^ genomes). Matching colour suggests consistent markers across experiments.
(XLS)

Table S7 **Summary of pod length as a covariate in analysis of pod strength (measured as rupture energy with pendulum test) with ID as random effect.**
(DOC)

Table S8 **Sequences, call rates, reproducibility, polymorphism information content of PAM (** ***in silico*** **DArT) markers identified using DArT-Seq.** Alignment of sequences with *B. rapa* and *B. oleracea* genomes is performed by Bowtie. Indices marked with * indicate alignments with bowtie and blast.
(XLSX)

Table S9 **Molecular markers associated with shatter resistance in a DH population from BLN2762/Surpass400 identified using Statistical Learning Machine method.** (supplementary methods). Highlighted markers are significantly associated with pod strength at *P* = 0.001. QTL detected using WGAIM are also shown (in italics with *‘Qrps.wwai’* suffix. Physical map position refers to the coordinates on the A and C sequenced genomes, and ‘0’ indicates no significant hit was found between query (GBS-Seq marker sequence) and the reference *B. rapa* and *B. oleracea* genomes.
(XLS)

Table S10 **Alignments between genetic regions that showed significant association with shatter resistance in the BLN2762/Surpass400 population with the sequenced genomes of** ***B. rapa*** **and** ***B. oleracea*** **.** Detailed description of candidate genes and their physical location of reference genomes are given. All markers which showed significant association with shatter resistance identified with SML, WGAIM and GWAS, were aligned with the A and C genomes. Only significant hits are given.
(XLSX)

Table S11 **Four haplotypes representing different** ***IND*** **and** ***SHP*** **allele combinations in a subset of DH lines of BLN2762/Surpass400 used for anatomical analysis.** ‘A’ and ‘B’ represent to BLN2762 and Surpass400 parental type alleles, respectively.
(DOC)

Table S12 **Favourable alleles (at consistent QTL,** **Table 1** **) showing their effects on shatter resistance in the DH lines.**
(XLS)

Table S13 **Genetic variation for shatter resistance in an F_2_ population derived from an interspecific cross between** ***B. napus*** **cv. Midas and** ***B. rapa*** **accession B-46.** Shatter resistance was measured using the cantilever test [28]. Figures given in parenthesis are coefficients of variation (%) within the intercross population.
(DOC)
