# Supplementary material for: Genome-Wide Delineation of Natural Variation for Pod Shatter Resistance in Brassica napus
Source: PLoS One. 2014 Jul 9;9(7):e101673. doi: 10.1371/journal.pone.0101673 (PMC4090071; doi:10.1371/journal.pone.0101673)

Supplemental figure 1: Transverse section of *B. carinata* accession ATC90239 pod at 40 days after anthesis visualised under fluorescence microscope

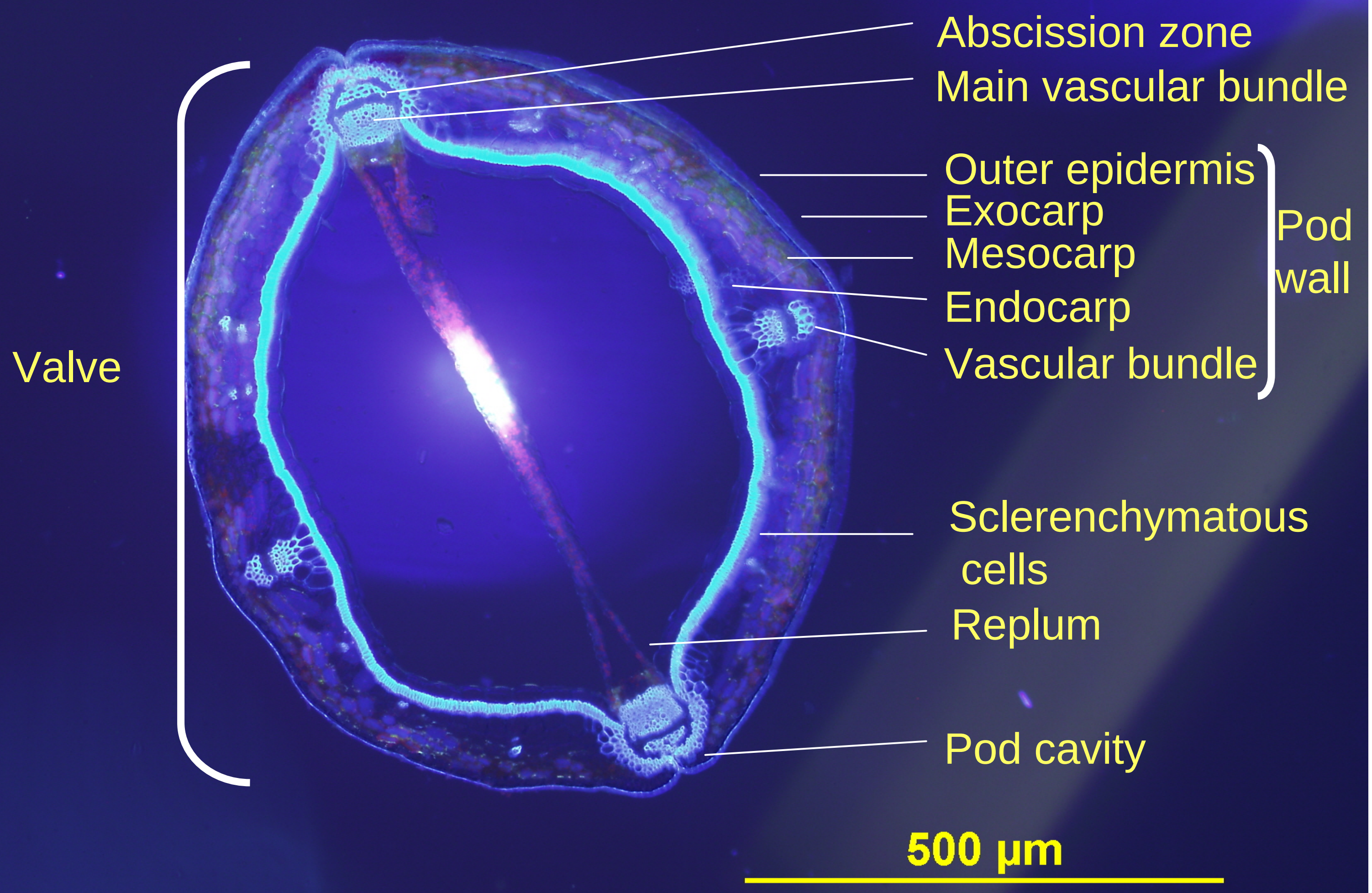

Supplement: Figure S1 — Transverse section of the B. carinata accession ATC90239 pod at 40 days after anthesis visualised under fluorescence. (PDF) [file pone.0101673.s001.pdf]
