# Supplementary material for: Genome-Wide Delineation of Natural Variation for Pod Shatter Resistance in Brassica napus
Source: PLoS One. 2014 Jul 9;9(7):e101673. doi: 10.1371/journal.pone.0101673 (PMC4090071; doi:10.1371/journal.pone.0101673)

Supplemental figure S2: Mapping of DArT-Seq^{TM} and non-DArT-Seq markers in relation to their recombination fractions and physical map positions on A and C genomes of *B. rapa* and *B. oleracea,* respectively.

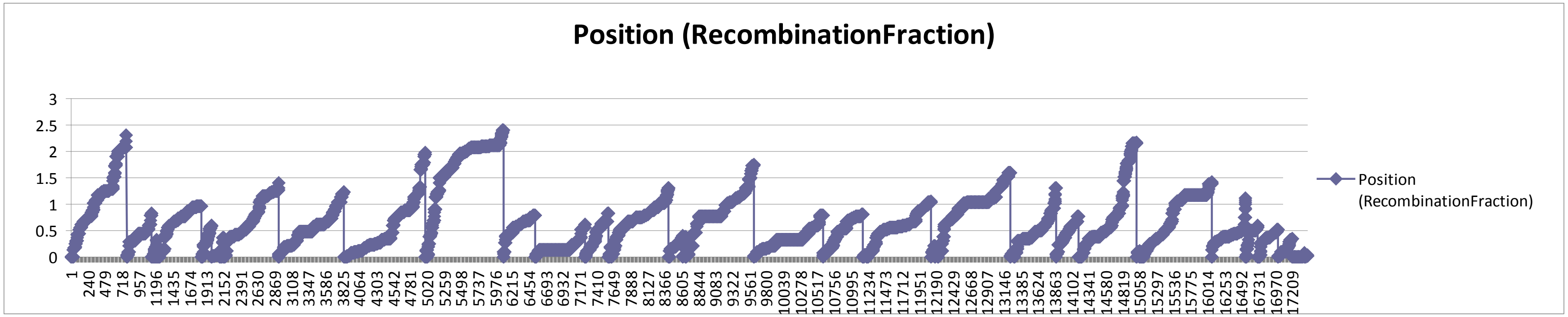

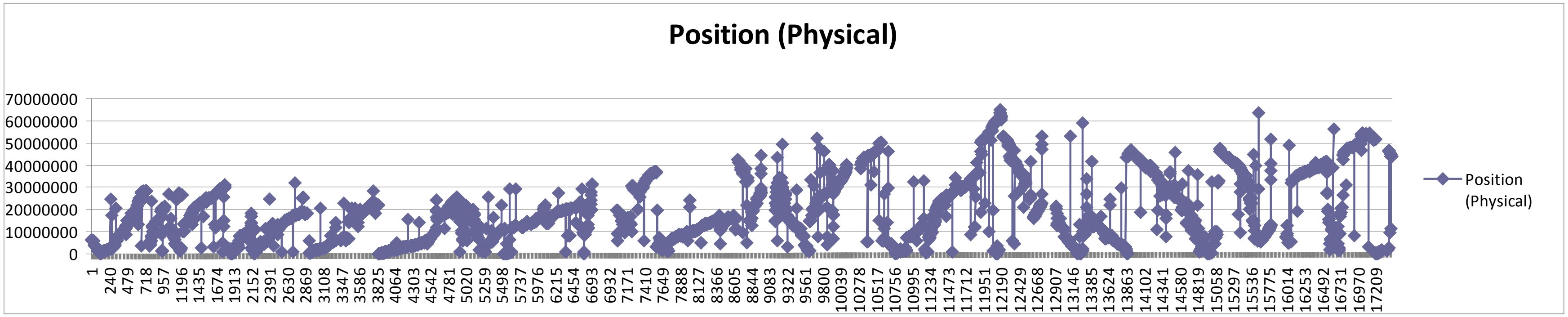

Supplement: Figure S2 — Mapping of DArT-Seq and non-DArT-Seq markers in relation to their recombination fractions and physical map positions on A and C genomes of B. rapa and B. oleracea , respectively. (PDF) [file pone.0101673.s002.pdf]
