# Supplementary material for: Genome-Wide Delineation of Natural Variation for Pod Shatter Resistance in Brassica napus
Source: PLoS One. 2014 Jul 9;9(7):e101673. doi: 10.1371/journal.pone.0101673 (PMC4090071; doi:10.1371/journal.pone.0101673)
Supplement: Figure S3 — Relationship between rupture energy among ∼200 diverse genotypes. (A: BIRDCAGE and FIELD experiments, p value = 4.44e-16, r = 0.57; B: SHT and GD experiments, p value = 2.22e-16, r = 0.55, and C: BIRDCAGE and FIELD experiments, p value = 8.88E-15, r = 0.55). ‘r’ indicates Pearson's product-moment correlation. (DOC) [file pone.0101673.s003.doc]

Supplemental Figure S3: Relationship between rupture energy among ~200 diverse genotypes (A: BIRDCAGE and FIELD experiments, p value = 4.44e-16, r = 0.57; B: SHT and GD experiments, p value = 2.22e-16, r = 0.55, and C: BIRDCAGE and FIELD experiments, p value = 8.88E-15, r = 0.55). ‘r’ indicates Pearson’s product-moment correlation.

(A)

(B)

(C)
