# Supplementary material for: Genome-Wide Delineation of Natural Variation for Pod Shatter Resistance in Brassica napus
Source: PLoS One. 2014 Jul 9;9(7):e101673. doi: 10.1371/journal.pone.0101673 (PMC4090071; doi:10.1371/journal.pone.0101673)
Supplement: Figure S4 — Phylogenetic analysis of DArT-Seq and non-DArT markers from different species of Brassica . The sidebars indicate the clades of different cultivars/species. The tree was constructed by the UPGMA method with Gower's distance. (DOC) [file pone.0101673.s004.doc]

**Supplemental Figure S4**: Phylogenetic analysis of DArT-Seq and non-DArT markers from different species of *Brassica*. The *sidebars* indicate the clades representing to different cultivars/species. The tree was constructed by the UPGMA method with Gower’s distance.


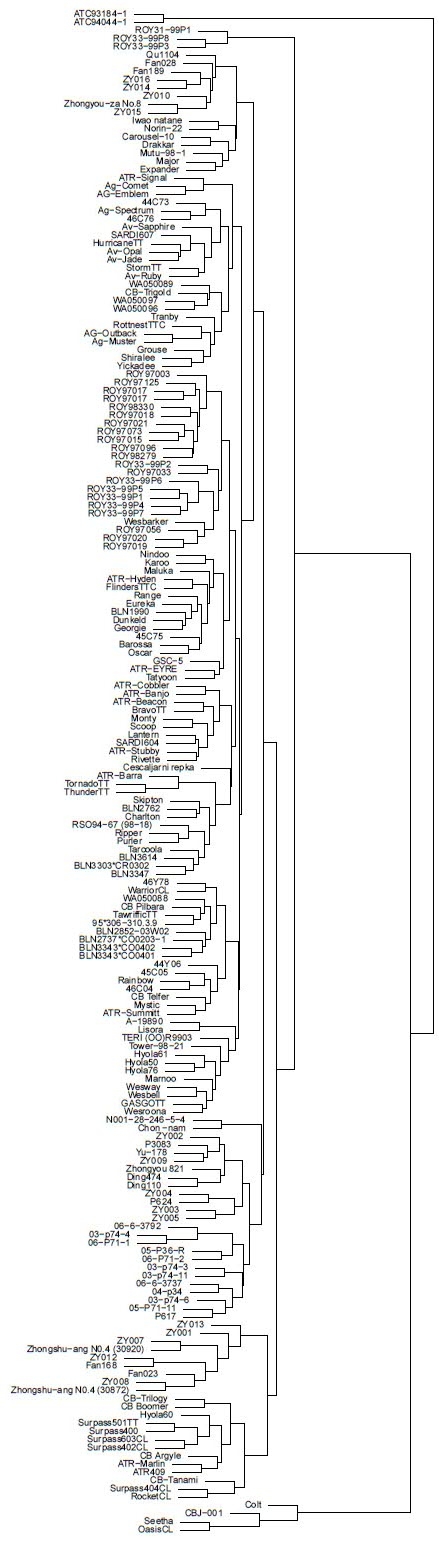


*B. carinata*

*B. napus*

*(China)*

*B. napus/*

*B. juncea*

*derivatives*

*B. napus*

*(China)*

*B. rapa*

*B. juncea*

I

II

III

IV

V
