# Supplementary material for: Genome-Wide Delineation of Natural Variation for Pod Shatter Resistance in Brassica napus
Source: PLoS One. 2014 Jul 9;9(7):e101673. doi: 10.1371/journal.pone.0101673 (PMC4090071; doi:10.1371/journal.pone.0101673)

Supplemental figure S5: Principal coordinate analysis was used to reveal overall genetic variation present in the genetic data of the diversity panel. The top 10 coordinates are shown in the bottom right panel along with the proportional of variance explained abbreviated as PAVE, along the y-axis (a) PCO plots of first three axis (x, y and z) in genotypes of *B. napus*, *B. rapa*, *B. carinata* and *B. juncea* and (b) PCO plots of first three axis (x, y and z) in genotypes of *B. napus*.

(a)

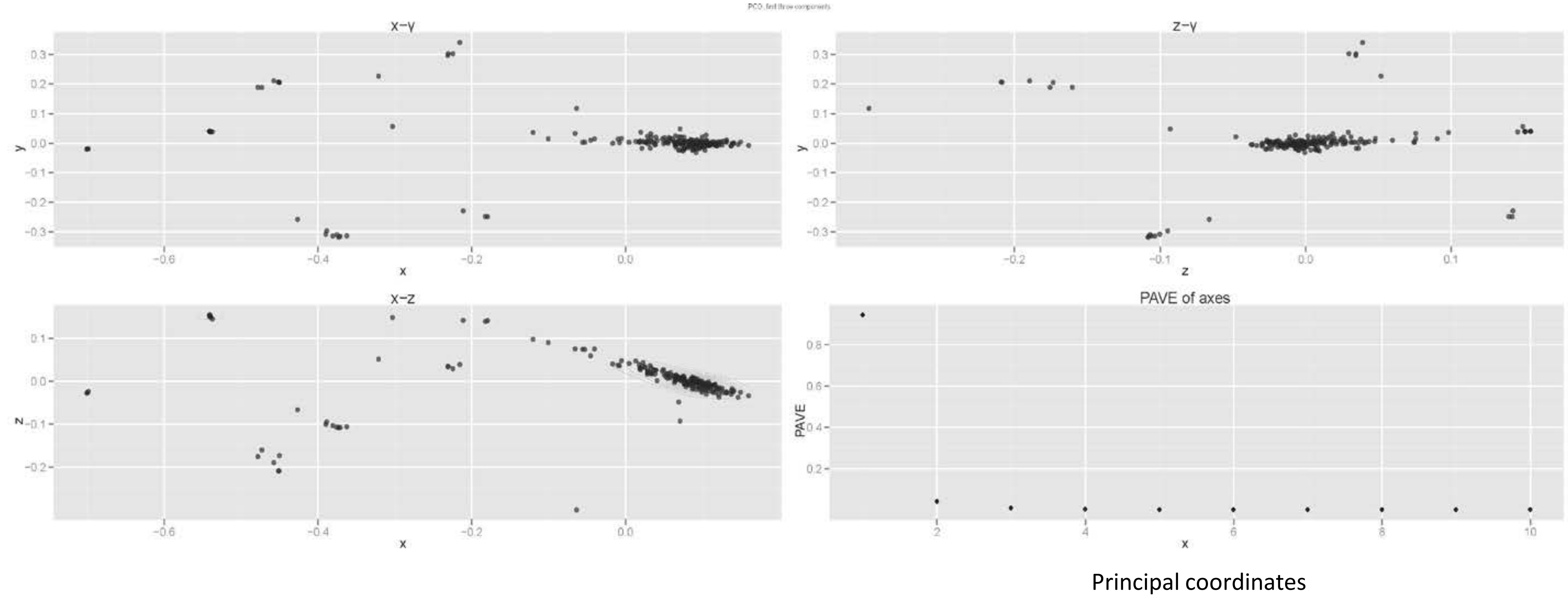

(b)

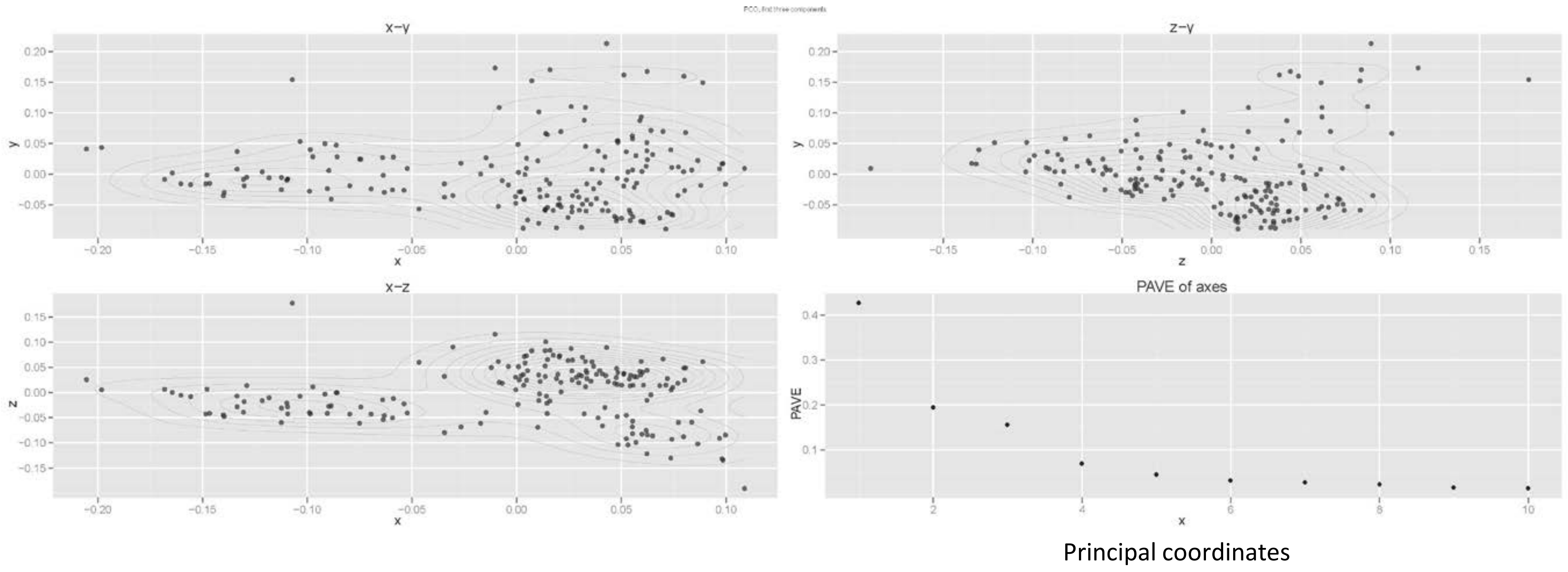

Supplement: Figure S5 — Principal coordinates analysis revealing overall genetic variation present in the genetic data of the diversity panel. The top 10 coordinates are shown in the bottom right panel along with the proportion of variance explained abbreviated as PAVE, on the y-axis. (a) PCO plots of first three axis (x, y and z) of B. napus, B. rapa, B. carinata and B. juncea genotypes and (b) PCO plots of first three axis (x, y and z) of B. napus. (PDF) [file pone.0101673.s005.pdf]
