# Supplementary material for: Genome-Wide Delineation of Natural Variation for Pod Shatter Resistance in Brassica napus
Source: PLoS One. 2014 Jul 9;9(7):e101673. doi: 10.1371/journal.pone.0101673 (PMC4090071; doi:10.1371/journal.pone.0101673)
Supplement: Figure S6 — Comparative analysis of marker intervals underlying QTL for shatter resistance on homoeologous chromosomes (a) A08/C08, (b) A03/C03 and A07/C06 in a B. napus DH population from BLN2762/Surpass400. Map positions are given to the left of the linkage groups (genetic distances are given in cM whereas, physical map distances are given in fractions (1/1,000,000th of the actual coordinates) of the B. rapa and B. oleracea scaffolds. Locus names are listed to the right. The QTL regions are marked with vertical bars to the left. Homologues are joined with solid lines between linkage groups. Organ identity and shatter resistance genes are given in italics. Candidate genes underlying the QTL (Table 1) are in bold. Query sequences were aligned with the genome scaffolds of B. rapa (ArAr genome) and B. oleracea (CoCo genome) and subsequently graphically represented using MapChart. QTL regions are connected with dotted lines. (DOC) [file pone.0101673.s006.doc]

**Supplemental Figure S6:** Comparative analysis of marker intervals underlying QTLs for shatter resistance on homoeologous chromosomes (a) A08/C08, (b) A03/C03 and A07/C06 in a *B. napus* DH population from BLN2762/Surpass400. Map positions are given to the *left* of the linkage groups (genetic distances are given in cM whereas, physical map distances are given in fractions (1/1,000,000^th^ of the actual coordinates) of the *B. rapa* and *B. oleracea* scaffolds. Locus names are listed to the *right*. The QTL regions are marked with vertical bars to the *left*. Homologues are joined with *solid* lines between linkage groups. Organ identity and shatter resistance genes are given *in italics*. Candidate genes underlying the QTLs (Table 1) are *in bold*. Query sequences were aligned with the genome scaffolds of *B. rapa* (A^r^A^r^ genome) and *B. oleracea* (C^o^C^o^ genome) and subsequently graphically represented using MapChart. QTL regions are connected with dotted lines.

(a)

*Qrps.wwai-A09*

*Qrps.wwai-C08c*

*Qrps.wwai-C08b*

*Qrps.wwai-C08a*

(b)

*Qrps.wwai-A03b*

*Qrps.wwai-C03*

( c)
