# Supplementary material for: Genome-Wide Delineation of Natural Variation for Pod Shatter Resistance in Brassica napus
Source: PLoS One. 2014 Jul 9;9(7):e101673. doi: 10.1371/journal.pone.0101673 (PMC4090071; doi:10.1371/journal.pone.0101673)
Supplement: Figure S7 — Homoeology between chromosomes A07 and C06 based on DArT sequences. Homologues are shown with solid lines. (DOC) [file pone.0101673.s007.doc]

Supplemental figure S7: Homoeology between chromosomes A07 and C06 based on DArT sequences. Homologues are shown with solid lines.
