# Supplementary material for: Genome-Wide Delineation of Natural Variation for Pod Shatter Resistance in Brassica napus
Source: PLoS One. 2014 Jul 9;9(7):e101673. doi: 10.1371/journal.pone.0101673 (PMC4090071; doi:10.1371/journal.pone.0101673)
Supplement: Table S1 — List of genotypes, their country of origin, and species used for genetic diversity analysis. (DOC) [file pone.0101673.s008.doc]

Supplemental Table S1: List of genotypes, their country of origin, and species used for genetic analysis and phenotyping for shatter resistance.

| Genotype | Country | *Species* | Source | Genetic status | Genotyping and Genetic Diversity | GWAS | Experiment* | | |
| --- | --- | --- | --- | --- | --- | --- | --- | --- | --- |
|  | of Origin |  |  |  |  |  | BIRDCAGE | SHT195 | GD200 |
| 6-06-3725 | Australia | *B. napus* | NSWDPI | Breeding line | N | N | N | Y | Y |
| 06-6-3737 | Australia | *B. napus* | NSWDPI | Breeding line | Y | Y | Y | N | Y |
| 06-6-3792 | Australia | *B. napus* | NSWDPI | Breeding line | Y | Y | Y | Y | Y |
| 03-p74-11 | China | *B. napus* | ACIAR | Breeding line | Y | Y | Y | Y | Y |
| 03-p74-3 | China | *B. napus* | ACIAR | Breeding line | Y | Y | Y | Y | Y |
| 03-p74-4 | China | *B. napus* | ACIAR | Breeding line | Y | Y | Y | Y | Y |
| 03-p74-6 | China | *B. napus* | ACIAR | Breeding line | Y | Y | Y | Y | Y |
| 04-p34 | China | *B. napus* | ACIAR | Breeding line | Y | Y | Y | Y | Y |
| 05-P36R | China | *B. napus* | ACIAR | Breeding line | Y | Y | Y | Y | Y |
| 05-P71-11 | China | *B. napus* | ACIAR | Breeding line | Y | Y | Y | Y | Y |
| 06-P71-1 | China | *B. napus* | ACIAR | Breeding line | Y | Y | Y | Y | Y |
| 06-P71-2 | China | *B. napus* | ACIAR | Breeding line | Y | Y | Y | Y | Y |
| 1B-5 | Unknown | *B. rapa* | ATCC, Horsham | Breeding line | N | N | N | Y | N |
| 44C73 | Australia | *B. napus* | Pioneer | Cultivar | Y | Y | Y | Y | Y |
| 44C79 | Australia | *B. napus* | PIONEER | Cultivar | N | N | N | Y | Y |
| 44Y06 | Australia | *B. napus* | Pioneer | Cultivar | Y | Y | Y | Y | Y |
| 45C05 | Australia | *B. napus* | Pioneer | Cultivar | Y | Y | Y | Y | Y |
| 45C75 | Australia | *B. napus* | Pioneer | Cultivar | Y | Y | Y | Y | Y |
| 45Y77 | Australia | *B. napus* | Pioneer | Cultivar | N | N | Y | Y | Y |
| 46C04 | Australia | *B. napus* | Pioneer | Cultivar | Y | Y | Y | Y | Y |
| 46C76 | Australia | *B. napus* | Pioneer | Cultivar | Y | Y | Y | Y | Y |
| 46Y78 | Australia | *B. napus* | Pioneer | Cultivar | Y | Y | Y | Y | Y |
| 46Y83 | Australia | *B. napus* | PIONEER | Cultivar | N | N | N | Y | Y |
| 95*306-310.3.9 | Unknown | *B. napus* | ATCC, Horsham | Breeding line | Y | Y | N | N | Y |
| 95-17033-2 | Unknown | *B. napus* | Unknown | Breeding line | N | N | Y | Y | Y |
| A-19890 | Poland? | *B. napus* | USDA | Breeding line | Y | Y | Y | Y | Y |
| AC-Excel | Canada | *B. napus* | ATCC, Horsham | Cultivar | N | N | N | N | Y |
| AC-Sunshine | Canada | *B. rapa* | ATCC, Horsham | Cultivar | N | N | Y | Y | Y |
| Ag-Comet | Australia | *B. napus* | Ag-Seed | Cultivar | Y | Y | Y | Y | Y |
| Ag-Drover | Australia | *B. napus* | Ag-Seed | Cultivar | N | N | N | Y | Y |
| AG-Emblem | Australia | *B. napus* | Ag-Seed | Cultivar | Y | Y | Y | Y | Y |
| Ag-Muster | Australia | *B. napus* | Ag-Seed | Cultivar | Y | Y | Y | Y | Y |
| AG-Outback | Australia | *B. napus* | Ag-Seed | Cultivar | Y | Y | Y | Y | Y |
| Ag-Spectrum | Australia | *B. napus* | Ag-Seed | Cultivar | Y | Y | Y | Y | Y |
| ATC93184-1 | Unknown | *B. carinata* | USDA | Breeding line | Y | N | Y | Y | Y |
| ATC94044-1 | Ethiopia | *B. carinata* | USDA | Breeding line | Y | N | Y | Y | Y |
| ATR409 | Australia | *B. napus* | Ag-Seed | Cultivar | Y | Y | Y | Y | Y |
| ATR-Banjo | Australia | *B. napus* | NUSEED | Cultivar | Y | Y | Y | Y | Y |
| ATR-Barra | Australia | *B. napus* | Ag-Seed | Cultivar | Y | Y | Y | Y | Y |
| ATR-Beacon | Australia | *B. napus* | AgVIC | Cultivar | Y | Y | Y | Y | Y |
| ATR-Cobbler | Australia | *B. napus* | Nugrain | Cultivar | Y | Y | Y | Y | Y |
| ATR-Eyre | Australia | *B. napus* | AgVIC | Cultivar | Y | Y | Y | N | N |
| ATR-Grace | Australia | *B. napus* | AgVIC | Cultivar | N | N | N | Y | N |
| ATR-Hyden | Australia | *B. napus* | Ag-Seed | Cultivar | Y | Y | Y | Y | Y |
| ATR-Marlin | Australia | *B. napus* | Ag-Seed | Cultivar | Y | Y | Y | Y | Y |
| ATR-Signal | Australia | *B. napus* | Nugrain | Cultivar | Y | Y | Y | Y | Y |
| ATR-Stubby | Australia | *B. napus* | Ag-Seed | Cultivar | Y | Y | Y | Y | Y |
| ATR-Summitt | Australia | *B. napus* | Ag-Seed | Cultivar | Y | Y | Y | Y | Y |
| AV-Garnet | Australia | *B. napus* | AgVIC | Cultivar | N | N | Y | Y | Y |
| Av-Jade | Australia | *B. napus* | Ag-Seed | Cultivar | Y | Y | Y | Y | Y |
| Av-Opal | Australia | *B. napus* | Ag-Seed | Cultivar | Y | Y | Y | Y | Y |
| Av-Ruby | Australia | *B. napus* | Ag-Seed | Cultivar | Y | Y | Y | Y | Y |
| Av-Sapphire | Australia | *B. napus* | Ag-Seed | Cultivar | Y | Y | Y | Y | Y |
| B-46 | India | *B. rapa* | ATCC, Horsham | Breeding line | N | N | N | Y | Y |
| Barossa | Australia | *B. napus* | NSWDPI | Cultivar | Y | Y | Y | N | Y |
| BLN1990 | Australia | *B. napus* | NSWDPI | Breeding line | Y | Y | Y | Y | Y |
| BLN2737*CO0203-1 | Australia | *B. napus* | NSWDPI | Breeding line | Y | Y | Y | Y | Y |
| BLN2762 | Australia | *B. napus* | NSWDPI | Breeding line | Y | Y | Y | Y | Y |
| BLN2852-03W02 | Australia | *B. napus* | NSWDPI | Breeding line | Y | Y | Y | Y | Y |
| BLN3303*CR0302 | Australia | *B. napus* | NSWDPI | Breeding line | Y | Y | Y | Y | Y |
| BLN3343*CO0401 | Australia | *B. napus* | NSWDPI | Breeding line | Y | Y | Y | Y | Y |
| BLN3343*CO0402 | Australia | *B. napus* | NSWDPI | Breeding line | Y | Y | Y | Y | Y |
| BLN3347 | Australia | *B. napus* | NSWDPI | Breeding line | Y | Y | Y | Y | Y |
| BLN3614 | Australia | *B. napus* | NSWDPI | Breeding line | Y | Y | Y | Y | Y |
| BravoTT | Australia | *B. napus* | Nuseed | Cultivar | Y | Y | Y | Y | Y |
| Carousel-10 | Europe | *B. napus* | Unknown | Selection | Y | Y | Y | Y | Y |
| CB-Argyle | Australia | *B. napus* | CBWA | Cultivar | Y | Y | Y | Y | Y |
| CB-Boomer | Australia | *B. napus* | CBWA | Cultivar | Y | Y | Y | Y | Y |
| CBJ-001 | China | *B. juncea* | ATCC, Horsham | Cultivar | Y | N | Y | Y | Y |
| CB-Pilbara | Australia | *B. napus* | NUSEED | Cultivar | Y | Y | Y | Y | Y |
| CB-Tanami | Australia | *B. napus* | CBWA | Cultivar | Y | Y | Y | Y | Y |
| CB-Telfer | Australia | *B. napus* | CBWA | Cultivar | Y | Y | Y | Y | Y |
| CB-Trigold | Australia | *B. napus* | CBWA | Cultivar | Y | Y | Y | Y | Y |
| CB-Trilogy | Australia | *B. napus* | CBWA | Cultivar | Y | Y | Y | Y | Y |
| Cescaljarni-repka | Korea | *B. napus* | ATCC, Horsham | Cultivar | Y | Y | Y | Y | Y |
| Charlton | Australia | *B. napus* | AgVIC | Cultivar | Y | Y | Y | Y | Y |
| Chon-nam | Korea | *B. napus* | ATCC, Horsham | Cultivar | Y | Y | Y | Y | Y |
| Colt | Unknown | *B. rapa* | Unknown | Cultivar | Y | N | N | N | Y |
| Ding110 | China | *B. napus* | ACIAR | Breeding line | Y | Y | Y | Y | Y |
| Ding474 | China | *B. napus* | ACIAR | Breeding line | Y | Y | Y | Y | Y |
| Drakkar | France | *B. napus* | INRA | Cultivar | Y | Y | Y | Y | Y |
| DS-17-D | Unknown | *B. rapa* | ATCC, Horsham | Breeding line | N | N | N | Y | Y |
| Dunkeld | Australia | *B. napus* | Ag-Seed | Cultivar | Y | Y | Y | Y | Y |
| Eureka | Australia | *B. napus* | NSWDPI | Cultivar | Y | Y | Y | Y | Y |
| Expander | Germany | *B. napus* | BGRC | Cultivar | Y | Y | Y | Y | Y |
| Fan023 | China | *B. napus* | ACIAR | Breeding line | Y | Y | Y | Y | Y |
| Fan028 | China | *B. napus* | ACIAR | Breeding line | Y | Y | Y | Y | Y |
| Fan168 | China | *B. napus* | ACIAR | Breeding line | Y | Y | Y | Y | Y |
| Fan189 | China | *B. napus* | ACIAR | Breeding line | Y | Y | Y | Y | Y |
| FlindersTTC | Australia | *B. napus* | Ag-Seed | Cultivar | Y | Y | Y | Y | Y |
| GASGOTT | Australia | *B. napus* | ATCC, Horsham | Cultivar | Y | Y | Y | Y | Y |
| Georgie | Australia | *B. napus* | NSWDPI | Cultivar | Y | Y | Y | N | N |
| Grouse | Australia | *B. napus* | NSWDPI | Cultivar | Y | Y | Y | Y | Y |
| GSC 5 | India | *B. napus* | Unknown | Breeding line | Y | Y | Y | Y | Y |
| HurricaneTT | Australia | *B. napus* | Pacific Seeds | Cultivar | Y | Y | Y | Y | Y |
| Hyola50 | Australia | *B. napus* | Pacific Seeds | Cultivar | Y | Y | Y | Y | Y |
| Hyola60 | Australia | *B. napus* | Pacific Seeds | Cultivar | Y | Y | Y | Y | Y |
| Hyola61 | Australia | *B. napus* | Pacific Seeds | Cultivar | Y | Y | Y | Y | Y |
| Hyola76 | Australia | *B. napus* | Pacific Seeds | Cultivar | Y | Y | Y | Y | Y |
| Iwao-natane | Japan | *B. napus* | Unknown | Cultivar | Y | Y | Y | Y | Y |
| Karoo | Australia | *B. napus* | AgVIC | Cultivar | Y | Y | Y | Y | Y |
| Lantern | Australia | *B. napus* | NSWDPI | Cultivar | Y | Y | Y | Y | Y |
| Lisora | Germany | *B. napus* | DSV | Cultivar | Y | Y | Y | N | Y |
| Major | France | *B. napus* | ATCC, Horsham | Cultivar | Y | Y | Y | Y | Y |
| Maluka | Australia | *B. napus* | NSWDPI | Cultivar | Y | Y | Y | Y | Y |
| Marnoo | Australia | *B. napus* | AgVIC | Cultivar | Y | Y | Y | Y | Y |
| Midas | Canada | *B. napus* | ATCC, Horsham | Cultivar | N | N | N | Y | Y |
| Monty | Australia | *B. napus* | NSWDPI | Cultivar | Y | Y | Y | Y | Y |
| Mutu-98-1 | Japan | *B. napus* | NSWDPI | Breeding line | Y | Y | Y | Y | Y |
| Mystic | Australia | *B. napus* | AgVIC | Cultivar | Y | Y | Y | Y | Y |
| N001-28-246-5-4 | Unknown | *B. napus* | ATCC, Horsham | Breeding line | Y | Y | N | N | N |
| Nindoo | Australia | *B. napus* | Unknown | Cultivar | Y | Y | Y | Y | Y |
| Norin-22 | Japan | *B. napus* | Unknown | Cultivar | Y | Y | Y | Y | Y |
| OasisCL | Australia | *B. juncea* | AgVIC | Cultivar | Y | N | Y | Y | Y |
| Oscar | Australia | *B. napus* | NSWDPI | Cultivar | Y | Y | Y | Y | Y |
| P3083 | China | *B. napus* | ACIAR | Breeding line | Y | Y | Y | Y | Y |
| P617 | China | *B. napus* | ACIAR | Breeding line | Y | Y | Y | Y | Y |
| P624 | China | *B. napus* | ACIAR | Breeding line | Y | Y | Y | Y | Y |
| Purler | Australia | *B. napus* | NSWDPI | Cultivar | Y | Y | Y | Y | Y |
| Qu1104 | China | *B. napus* | ACIAR | Breeding line | Y | Y | Y | Y | Y |
| Rafal | France | *B. napus* | ATCC, Horsham | Cultivar | N | N | N | Y | Y |
| Rainbow | Australia | *B. napus* | AgVIC | Cultivar | Y | Y | Y | Y | Y |
| Range | Australia | *B. napus* | Ag-Seed | Cultivar | Y | Y | Y | Y | Y |
| Ripper | Australia | *B. napus* | NSWDPI | Cultivar | Y | Y | Y | Y | Y |
| Rivette | Australia | *B. napus* | NSWDPI | Cultivar | Y | Y | Y | Y | Y |
| RocketCL | Australia | *B. napus* | Pacific Seeds | Cultivar | Y | Y | Y | Y | Y |
| RottnestTTC | Australia | *B. napus* | Nuseed | Cultivar | Y | Y | Y | Y | Y |
| ROY31-99P1 | Australia | *B. napus* | DAFWA | Breeding line | Y | Y | Y | Y | Y |
| ROY33-99P1 | Australia | *B. napus* | DAFWA | Breeding line | Y | Y | Y | Y | Y |
| ROY33-99P2 | Australia | *B. napus* | DAFWA | Breeding line | Y | Y | Y | Y | Y |
| ROY33-99P3 | Australia | *B. napus* | DAFWA | Breeding line | Y | Y | Y | Y | Y |
| ROY33-99P4 | Australia | *B. napus* | DAFWA | Breeding line | Y | Y | Y | Y | Y |
| ROY33-99P5 | Australia | *B. napus* | DAFWA | Breeding line | Y | Y | Y | Y | Y |
| ROY33-99P6 | Australia | *B. napus* | DAFWA | Breeding line | Y | Y | Y | Y | Y |
| ROY33-99P7 | Australia | *B. napus* | DAFWA | Breeding line | Y | Y | Y | Y | Y |
| ROY33-99P8 | Australia | *B. napus* | DAFWA | Breeding line | Y | Y | Y | Y | Y |
| ROY97003 | Australia | *B. napus* | DAFWA | Breeding line | Y | Y | Y | Y | Y |
| ROY97015 | Australia | *B. napus* | DAFWA | Breeding line | Y | Y | Y | Y | Y |
| ROY97017-1 | Australia | *B. napus* | DAFWA | Breeding line | Y | Y | Y | N | Y |
| ROY97017-2 | Australia | *B. napus* | DAFWA | Breeding line | Y | Y | Y | N | Y |
| ROY97018 | Australia | *B. napus* | DAFWA | Breeding line | Y | Y | Y | Y | Y |
| ROY97019 | Australia | *B. napus* | DAFWA | Breeding line | Y | Y | Y | N | N |
| ROY97020 | Australia | *B. napus* | DAFWA | Breeding line | Y | Y | Y | Y | Y |
| ROY97021 | Australia | *B. napus* | DAFWA | Breeding line | Y | Y | Y | Y | Y |
| ROY97033 | Australia | *B. napus* | DAFWA | Breeding line | Y | Y | Y | Y | Y |
| ROY97056 | Australia | *B. napus* | DAFWA | Breeding line | Y | Y | Y | Y | Y |
| ROY97073 | Australia | *B. napus* | DAFWA | Breeding line | Y | Y | Y | N | N |
| ROY97096 | Australia | *B. napus* | DAFWA | Breeding line | Y | Y | Y | Y | Y |
| ROY97125 | Australia | *B. napus* | DAFWA | Breeding line | Y | Y | Y | Y | Y |
| ROY98279 | Australia | *B. napus* | DAFWA | Breeding line | Y | Y | Y | Y | Y |
| ROY98330 | Australia | *B. napus* | DAFWA | Breeding line | Y | Y | Y | Y | Y |
| RSO94-67(98-18) | Unknown | *B. napus* | Unknown | Breeding line | Y | Y | Y | Y | Y |
| RT057 | Unknown | *B. napus* | ATCC, Horsham | Breeding line | N | N | N | Y | Y |
| RT076 | Unknown | *B. napus* | ATCC, Horsham | Breeding line | N | N | N | Y | Y |
| SaharaCL | Australia | *B. juncea* | VITERRA | Cultivar | N | N | Y | Y | Y |
| SARDI604 | Australia | *B. napus* | SARDI | Cultivar | Y | Y | Y | Y | Y |
| SARDI607 | Australia | *B. napus* | SARDI | Cultivar | Y | Y | Y | Y | Y |
| Scoop | Australia | *B. napus* | NSWDPI | Cultivar | Y | Y | Y | Y | Y |
| Seetha | India | *B. juncea* | Unknown | Cultivar | Y | N | Y | Y | Y |
| Shiralee | Australia | *B. napus* | NSWDPI | Cultivar | Y | Y | Y | Y | Y |
| Skipton | Australia | *B. napus* | NSWDPI | Cultivar | Y | Y | Y | Y | Y |
| StormTT | Australia | *B. napus* | Pacific seeds | Cultivar | Y | Y | Y | Y | Y |
| Surpass400 | Australia | *B. napus* | Pacific Seeds | Cultivar | Y | Y | Y | Y | Y |
| Surpass402CL | Australia | *B. napus* | Pacific Seeds | Cultivar | Y | Y | Y | Y | Y |
| Surpass404CL | Australia | *B. napus* | Pacific Seeds | Cultivar | Y | Y | Y | Y | Y |
| Surpass501TT | Australia | *B. napus* | Pacific Seeds | Cultivar | Y | Y | Y | Y | Y |
| Surpass603CL | Australia | *B. napus* | Pacific Seeds | Cultivar | Y | Y | Y | Y | Y |
| Tarcoola | Australia | *B. napus* | NSWDPI | Cultivar | Y | Y | Y | Y | Y |
| Tatyoon | Australia | *B. napus* | AgVIC | Cultivar | Y | Y | Y | Y | Y |
| TawrifficTT | Australia | *B. napus* | Nugrain | Cultivar | Y | Y | Y | Y | Y |
| TERI(OO)R9903 | Australia | *B. napus* | India | Breeding line | Y | Y | Y | Y | Y |
| ThunderTT | Australia | *B. napus* | Pacific Seeds | Cultivar | Y | Y | Y | Y | Y |
| Torch | Canada | *B. rapa* | ATCC, Horsham | Cultivar | N | N | N | Y | Y |
| TornadoTT | Australia | *B. napus* | Pacific Seeds | Cultivar | Y | Y | Y | Y | Y |
| Tower-98-21 | Canada | *B. napus* | ATCC, Horsham | Breeding line | Y | Y | Y | Y | Y |
| Tranby | Australia | *B. napus* | WADA | Cultivar | Y | Y | Y | Y | Y |
| Urvashi | India | *B. juncea* | ACIAR | Cultivar | N | N | N | Y | Y |
| WA050085 | Australia | *B. napus* | DAFWA | Breeding line | N | N | N | Y | N |
| WA050088 | Australia | *B. napus* | DAFWA | Breeding line | Y | Y | Y | N | Y |
| WA050089 | Australia | *B. napus* | DAFWA | Breeding line | Y | Y | N | Y | Y |
| WA050095 | Australia | *B. napus* | DAFWA | Breeding line | N | N | N | Y | N |
| WA050096 | Australia | *B. napus* | DAFWA | Breeding line | Y | Y | Y | Y | Y |
| WA050097 | Australia | *B. napus* | DAFWA | Breeding line | Y | Y | Y | N | Y |
| WarriorCL | Australia | *B. napus* | NSWDPI | Cultivar | Y | Y | Y | Y | Y |
| Wesbarker | Australia | *B. napus* | AgWA | Cultivar | Y | Y | Y | Y | Y |
| Wesbell | Australia | *B. napus* | AgWA | Cultivar | Y | Y | Y | Y | Y |
| Wesreo | Australia | *B. napus* | AgWA | Cultivar | N | N | Y | Y | Y |
| Wesroona | Australia | *B. napus* | AgWA | Cultivar | Y | Y | Y | Y | Y |
| Westar | Canada | *B. napus* | ATCC, Horsham | Cultivar | N | N | Y | Y | Y |
| Wesway | Australia | *B. napus* | AgWA | Cultivar | Y | Y | Y | Y | Y |
| Yickadee | Australia | *B. napus* | NSWDPI | Cultivar | Y | Y | Y | Y | Y |
| Yu-178 | China | *B. napus* | ACIAR | Cultivar | Y | Y | Y | Y | Y |
| Zhongshu-angN0.4(30872) | China | *B. napus* | ACIAR | Breeding line | Y | Y | Y | Y | Y |
| Zhongshu-angN0.4(30920) | China | *B. napus* | ACIAR | Breeding line | Y | Y | Y | Y | Y |
| Zhongyou821 | China | *B. napus* | ACIAR | Cultivar | Y | Y | Y | Y | Y |
| Zhongyou-angNo.8 | China | *B. napus* | ACIAR | Breeding line | Y | Y | Y | Y | Y |
| ZY001 | China | *B. napus* | Unknown | Breeding line | Y | Y | Y | Y | Y |
| ZY002 | China | *B. napus* | Unknown | Breeding line | Y | Y | Y | Y | Y |
| ZY003 | China | *B. napus* | Unknown | Breeding line | Y | Y | Y | Y | Y |
| ZY004 | China | *B. napus* | Unknown | Breeding line | Y | Y | Y | Y | Y |
| ZY005 | China | *B. napus* | Unknown | Breeding line | Y | Y | Y | Y | Y |
| ZY007 | China | *B. napus* | Unknown | Breeding line | Y | Y | Y | Y | Y |
| ZY008 | China | *B. napus* | Unknown | Breeding line | Y | Y | Y | Y | Y |
| ZY009 | China | *B. napus* | Unknown | Breeding line | Y | Y | Y | Y | Y |
| ZY010 | China | *B. napus* | Unknown | Breeding line | Y | Y | Y | Y | Y |
| ZY012 | China | *B. napus* | Unknown | Breeding line | Y | Y | Y | Y | Y |
| ZY013 | China | *B. napus* | Unknown | Breeding line | Y | Y | Y | Y | Y |
| ZY014 | China | *B. napus* | Unknown | Breeding line | Y | Y | Y | Y | Y |
| ZY015 | China | *B. napus* | Unknown | Breeding line | Y | Y | Y | Y | Y |
| ZY016 | China | *B. napus* | Unknown | Breeding line | Y | Y | Y | Y | Y |

* Y: Yes, N: No
