# Supplementary material for: Genome-Wide Delineation of Natural Variation for Pod Shatter Resistance in Brassica napus
Source: PLoS One. 2014 Jul 9;9(7):e101673. doi: 10.1371/journal.pone.0101673 (PMC4090071; doi:10.1371/journal.pone.0101673)
Supplement: Table S5 — Predicted means of the parental lines of BLN2762/Surpass400 DH population used for phenotyping. Frequency distribution of DH lines is shown in Figure 1. Transformations are square-root of shatter and natural logarithm of RELSQ. (DOC) [file pone.0101673.s012.doc]

Supplemental Table S5: Predicted means of the parental lines of BLN2762/ Surpass400 DH population used for phenotyping. Frequency distribution of DH lines is shown in Figure 1. Transformations are square root of shatter and natural logarithm of RELSQ.

| Experiment | Genotype | Predicted Mean (Mj) | Standard Error |
| --- | --- | --- | --- |
| SHT11 | BLN2762 | 2.055590 | 0.3308059 |
|  | DH | 1.743651 | 0.0294426 |
|  | Surpass400 | 1.479838 | 0.3040383 |
| RELSQ11 | BLN2762 | 0.4721451 | 0.4758995 |
|  | DH | 0.1780248 | 0.0419888 |
|  | Surpass400 | -0.2912711 | 0.4506892 |
| SHT12 | BLN2762 | 1.667420 | 0.26069693 |
|  | DH | 1.802964 | 0.03046708 |
|  | Surpass400 | 1.710282 | 0.26012763 |
| RELSQ12 | BLN2762 | 0.1381022 | 0.4366650 |
|  | DH | 0.2526231 | 0.0608044 |
|  | Surpass400 | 0.1935702 | 0.4368411 |
| WWSHT12 | BLN2762 | 1.940280 | 0.24480448 |
|  | DH | 1.878485 | 0.02840718 |
|  | Surpass400 | 1.759387 | 0.24522792 |
| WWRELSQ12 | BLN2762 | 0.1903534 | 0.3732021 |
|  | DH | 0.0843909 | 0.0567100 |
|  | Surpass400 | -0.1167410 | 0.3736447 |
