# Supplementary material for: Genome-Wide Delineation of Natural Variation for Pod Shatter Resistance in Brassica napus
Source: PLoS One. 2014 Jul 9;9(7):e101673. doi: 10.1371/journal.pone.0101673 (PMC4090071; doi:10.1371/journal.pone.0101673)
Supplement: Table S7 — Summary of pod length as a covariate in analysis of pod strength (measured as rupture energy with pendulum test) with ID as random effect. (DOC) [file pone.0101673.s014.doc]

Supplemental Table S7: Summary of results of a variance component analysis pod length as covariate in analysis of pod strength (measured as rupture energy with pendulum test) with ID as random effect.

| Experiment | Pod Length | | ID |
| --- | --- | --- | --- |
|  | Covariate | Standard Error | Percent variability |
| DH population from BLN2762/Surpass400 | | | |
| SHT11 | .05137 | .004772 | 27.0 |
| SHT12 | .03344 | .004947 | 18.6 |
| SHT12WW | .02085 | .005307 | 18.0 |
|  | | | |
| Genetic diversity set used for GWAS analysis | | | |
| Birdcage | .03818 | .003379 | 16.6 |
| SHT195 | .03679 | .003092 | 18.0 |
| GD200 | .06226 | .004107 | 23.6 |
