# Supplementary material for: Genome-Wide Delineation of Natural Variation for Pod Shatter Resistance in Brassica napus
Source: PLoS One. 2014 Jul 9;9(7):e101673. doi: 10.1371/journal.pone.0101673 (PMC4090071; doi:10.1371/journal.pone.0101673)
Supplement: Table S11 — Four haplotypes representing different IND and SHP allele combinations in a subset of DH lines of BLN2762/Surpass400 used for anatomical analysis. ‘A’ and ‘B’ represent to BLN2762 and Surpass400 parental type alleles, respectively. (DOC) [file pone.0101673.s018.doc]

| **No.** | **ID of DH from BLN2762/Surpass** | ***INDEHISCENT***  **gene based marker** | ***SHATTERPROOF***  **gene based marker** |
| --- | --- | --- | --- |
| **Haplotype 1** | | | |
| 1 | C6665 | B | A |
| 2 | C6983 | B | A |
| 3 | C6850 | B | A |
| 4 | C7148 | B | A |
| 5 | C6660 | B | A |
| 6 | C7133 | B | A |
| 7 | C6668 | B | A |
| 8 | C6672 | B | A |
| 9 | C7139 | B | A |
| 10 | C6840 | B | A |
| **Haplotype 2** | | | |
| 11 | C6827 | A | A |
| 12 | C6823 | A | A |
| 13 | C6649 | A | A |
| 14 | C7252 | A | A |
| 15 | C6837 | A | A |
| 16 | C7237 | A | A |
| 17 | C6842 | A | A |
| 18 | C7137 | A | A |
| 19 | C7149 | A | A |
| 20 | C7261 | A | A |
| **Haplotype 3** | | | |
| 21 | C6656 | B | B |
| 22 | C7136 | B | B |
| 23 | C6651 | B | B |
| 24 | C6674 | B | B |
| 25 | C6881 | B | B |
| 26 | C6832 | B | B |
| 27 | C7124 | B | B |
| 28 | C7138 | B | B |
| 29 | C6675 | B | B |
| 30 | C7152 | B | B |
| **Haplotype 4** | | | |
| 31 | C6883 | A | B |
| 32 | C7250 | A | B |
| 33 | C6826 | A | B |
| 34 | C6836 | A | B |
| 35 | C6845 | A | B |
| 36 | C7129 | A | B |
| 37 | C6824 | A | B |
| 38 | C6846 | A | B |
| 39 | C6676 | A | B |
| 40 | C7128 | A | B |

Supplemental Table S11: Four haplotypes representing different *IND* and *SHP* allele combinations in a subset of DH lines of BLN2762/Surpass400 used for anatomical analysis. ‘A’ and ‘B’ represent to BLN2762 and Surpass400 parental type alleles, respectively.
