# Supplementary material for: Genome-Wide Delineation of Natural Variation for Pod Shatter Resistance in Brassica napus
Source: PLoS One. 2014 Jul 9;9(7):e101673. doi: 10.1371/journal.pone.0101673 (PMC4090071; doi:10.1371/journal.pone.0101673)
Supplement: Table S13 — Genetic variation for shatter resistance in an F2 population derived from an interspecific cross between B. napus cv. Midas and B. rapa accession B-46. Shatter resistance was measured using the cantilever test [28]. Figures given in parenthesis are coefficients of variation (%) within the intercross population. (DOC) [file pone.0101673.s020.doc]

Supplemental Table S13: Genetic variation for shatter resistance in an F_2_ population derived from an interspecific cross between *B. napus* cv. Midas and *B. rapa* accession B-46. Shatter resistance was measured using cantilever test [45]. Figures given in parenthesis are coefficient of variation (%) within the intercross population.

| Genotype | Energy ± SE(mJ) | RELSQ ± SE(mj mm ^-1^) |
| --- | --- | --- |
| Midas | 0.11 ± 0.01 | 2.09 ± 0.24 |
| B-46 | 4.52 ± 0.48 | 89.46 ± 10.39 |
| Midas/B-46 | 0.04 – 0.66 (77) | 0.95- 23.99 (91) |
